# Supplementary material for: Decoding the mechanisms underlying cell-fate decision-making during stem cell differentiation by random circuit perturbation
Source: J R Soc Interface. 2020 Aug 12;17(169):20200500. doi: 10.1098/rsif.2020.0500 (PMC7482558; doi:10.1098/rsif.2020.0500)
Supplement: Stem cell_SI_Final.docx [file rsif20200500supp1.docx]

**Supplementary Information**

**Decoding the mechanisms underlying cell-fate decision-making during stem cell differentiation by random circuit perturbation**

Bin Huang^1*^, Mingyang Lu^2*^, Madeline Galbraith^1,3^, Herbert Levine^1,4,5,#^, Jose N. Onuchic^1,3,6,7,#^, Dongya Jia^1,#^

^1^Center for Theoretical Biological Physics, Rice University, Houston, TX 77005, USA

^2^The Jackson Laboratory, 600 Main St, Bar Harbor, ME 04609, USA

^3^Department of Physics and Astronomy, Rice University, Houston, TX 77005, USA

^4^Department of Bioengineering, Northeastern University, Boston, MA 02115, USA

^5^Department of Physics, Northeastern University, Boston, MA 02115, USA

^6^Department of Chemistry, Rice University, Houston, TX 77005, USA

^7^Department of Bioengineering, Rice University, Houston, TX 77005, USA

*These authors contributed equally to this work.

^#^To whom correspondence can be addressed:

D.J. ([dj9@rice.edu](mailto:dj9@rice.edu)), H.L. ([h.levine@northeastern.edu](mailto:h.levine@northeastern.edu)) and J.N.O. ([jonuchic@rice.edu](mailto:jonuchic@rice.edu)).

**Supplementary Notes**

1. **Mathematical modeling of the Stemness GRN by RACIPE**

Based on previous studies, we constructed a core gene regulatory circuit governing the decision making of mouse stem cell differentiation. The circuit is composed of eight transcription factors (Oct4, Cdx2, etc.), a protein complex (OCT4-SOX2) and 25 regulatory links between them (**figure 2a**). Due to the complexity of the circuit, the operative function of this circuit remains elusive by traditional modeling approaches. The dynamics of the stem cell circuit can be described by the deterministic rate equations as follows:

$$\dot{X_{1}}=\frac{G_{X_{1}}}{\lambda_{X_{1}X_{1}}^{+}*\lambda_{X_{7}X_{1}}^{+}}{*H}^{S}\left( X_{1}, X_{1}X_{10},n_{X_{1}X_{1}},\lambda_{X_{1}X_{1}}^{+} \right){*H}^{S}\left( X_{5}, X_{5}X_{10},n_{X_{5}X_{1}},\lambda_{X_{5}X_{1}}^{-} \right){*H}^{S}\left( X_{7}, X_{7}X_{10},n_{X_{7}X_{1}},\lambda_{X_{7}X_{1}}^{+} \right)*H^{S}\left( X_{8}, X_{8}X_{10},n_{X_{8}X_{1}},\lambda_{X_{8}X_{1}}^{-} \right)- K_{X_{1}}*X_{1}$$

$$\dot{X_{2}}=\frac{G_{X_{2}}}{\lambda_{X_{1}X_{2}}^{+}*\lambda_{X_{3}X_{2}}^{+}}{*H}^{S}\left( X_{1}, X_{1}X_{20},n_{X_{1}X_{2}},\lambda_{X_{1}X_{2}}^{+} \right){*H}^{S}\left( X_{3}, X_{3}X_{20},n_{X_{3}X_{2}},\lambda_{X_{3}X_{2}}^{+} \right)- K_{X_{2}}*X_{2}$$

$$\dot{X_{3}}=\frac{G_{X_{3}}}{\lambda_{X_{3}X_{3}}^{+}}{*H}^{S}\left( X_{3}, X_{3}X_{30},n_{X_{3}X_{3}},\lambda_{X_{3}X_{3}}^{+} \right){*H}^{S}\left( X_{5}, X_{5}X_{30},n_{X_{5}X_{3}},\lambda_{X_{5}X_{3}}^{-} \right){*H}^{S}\left( X_{7}, X_{7}X_{30},n_{X_{7}X_{3}},\lambda_{X_{7}X_{3}}^{-} \right)- K_{X_{3}}*X_{3}$$

$$\dot{X_{4}}=\frac{G_{X_{4}}}{\lambda_{X_{5}X_{4}}^{+}*\lambda_{X_{7}X_{4}}^{+}*\lambda_{X_{9}X_{4}}^{+}}{*H}^{S}\left( X_{5}, X_{5}X_{40},n_{X_{5}X_{4}},\lambda_{X_{5}X_{4}}^{+} \right){*H}^{S}\left( X_{7}, X_{7}X_{40},n_{X_{7}X_{4}},\lambda_{X_{7}X_{4}}^{+} \right){*H}^{S}\left( X_{9}, X_{9}X_{40},n_{X_{9}X_{4}},\lambda_{X_{9}X_{4}}^{+} \right)- K_{X_{4}}*X_{4}$$

$$\dot{X_{5}}=\frac{G_{X_{5}}}{\lambda_{X_{4}X_{5}}^{+}*\lambda_{X_{6}X_{5}}^{+}*\lambda_{X_{8}X_{5}}^{+}}{*H}^{S}\left( X_{1}, X_{1}X_{50},n_{X_{1}X_{5}},\lambda_{X_{1}X_{5}}^{-} \right){*H}^{S}\left( X_{3}, X_{3}X_{50},n_{X_{3}X_{5}},\lambda_{X_{3}X_{5}}^{-} \right){*H}^{S}\left( X_{4}, X_{4}X_{50},n_{X_{4}X_{5}},\lambda_{X_{4}X_{5}}^{+} \right)*H^{S}\left( X_{5}, X_{5}X_{50},n_{X_{5}X_{5}},\lambda_{X_{5}X_{5}}^{-} \right){*H}^{S}\left( X_{6}, X_{6}X_{50},n_{X_{6}X_{5}},\lambda_{X_{6}X_{5}}^{+} \right){*H}^{S}\left( X_{8}, X_{8}X_{50},n_{X_{8}X_{5}},\lambda_{X_{8}X_{5}}^{+} \right) - K_{X_{5}}*X_{5}$$

$$\dot{X_{6}}=\frac{G_{X_{6}}}{\lambda_{X_{5}X_{6}}^{+}}{*H}^{S}\left( X_{5}, X_{5}X_{60},n_{X_{5}X_{6}},\lambda_{X_{5}X_{6}}^{+} \right)- K_{X_{6}}*X_{6}$$

$$\dot{X_{7}}=\frac{G_{X_{7}}}{\lambda_{X_{8}X_{7}}^{+}}{*H}^{S}\left( X_{2}, X_{2}X_{70},n_{X_{2}X_{7}},\lambda_{X_{2}X_{7}}^{-} \right){*H}^{S}\left( X_{3}, X_{3}X_{70},n_{X_{3}X_{7}},\lambda_{X_{3}X_{7}}^{-} \right){*H}^{S}\left( X_{8}, X_{8}X_{70},n_{X_{8}X_{7}},\lambda_{X_{8}X_{7}}^{+} \right)- K_{X_{7}}*X_{7}$$

$$\dot{X_{8}}=\frac{G_{X_{8}}}{\lambda_{X_{7}X_{8}}^{+}*\lambda_{X_{9}X_{8}}^{+}}{*H}^{S}\left( X_{7}, X_{7}X_{80},n_{X_{7}X_{8}},\lambda_{X_{7}X_{8}}^{+} \right){*H}^{S}\left( X_{9}, X_{9}X_{80},n_{X_{9}X_{8}},\lambda_{X_{9}X_{8}}^{+} \right)- K_{X_{8}}*X_{8}$$

$$\dot{X_{9}}=\frac{G_{X_{9}}}{\lambda_{X_{8}X_{9}}^{+}}{*H}^{S}\left( X_{8}, X_{8}X_{90},n_{X_{8}X_{9}},\lambda_{X_{8}X_{9}}^{+} \right)- K_{X_{9}}*X_{9}$$

where $G_{i}$ represents the maximum production rate of gene i, and $k_{i}$ represents the degradation rate of gene i. The transcriptional regulation between the TFs A and B is modeled by a non-linear shifted Hill function $H^{S}(B, {BA}_{0},n_{BA},\lambda_{BA})$ defined as $H^{S}\left( B, {BA}_{0},n_{BA},\lambda_{BA} \right)=\frac{1+\lambda_{BA}\left( B/{{BA}_{0}} \right)^{n_{BA}}}{1+\left( B/{{BA}_{0}} \right)^{n_{BA}}}$, where ${BA}_{0}$ is the threshold level of TF B, $n_{BA}$ is the Hill coefficient and $\lambda_{BA}$ represents the maximum fold-change of the level of TF A caused by the TF B. For inhibitory regulation, $\lambda_{BA}\in(0.01,1)$, referred to as $\lambda_{BA}^{-}$ and for excitatory regulation, $\lambda_{BA}\in(1, 100)$, referred to as $\lambda_{BA}^{+}.$ $X_{n}$ (n = 1, 2 … 9) stands for the expression levels of Gata6, Gcnf, Cdx2, Klf4, Nanog, Pbx1, Oct4, OCT4-SOX2 complex and Sox2, respectively. Here, the OCT4-SOX2 complex is modeled in the same way as the other genes.

The dynamical behavior of the stem cell circuit was further analyzed by using our recently developed algorithm, RACIPE. Instead of finding a representative set of kinetic parameters, we randomly generated 10,000 RACIPE models, each of which corresponds to a distinct set of kinetic parameters. The purpose of the RACIPE-based approach is to identify robust dynamical features of the circuit that are not observed from only a small portion of models. For each RACIPE model, we found all possible stable steady states by numerical simulations. Here, we simulated the above-mentioned ordinary differential equations (ODEs) starting from a random initial condition and until the system reaches to a steady state. We repeated the calculations 1,000 times with different initial conditions to thoroughly explore the solution space for each model. From the 1,000 steady state solutions, we identified the number of distinct stable states, and the corresponding gene expression profiles. RACIPE was applied on the stem cell circuit with 10 independent repeats, and we identified the converged probability distribution of the number of stable states for each model (**figure 2b**). We found there are some rare occasions (<1%) to have a RACIPE model with more than six coexisting stable steady states. Since they are not statistically significant, we excluded these data from further analysis. The circuit also has ~ 2% chance to have oscillatory or chaotic dynamics, which are also excluded from the analysis.

1. **Mathematical modeling of the Stemness GRN by generalized RACIPE which includes the binding/unbinding processes between Oct4 and Sox2, also referred to as RACIPE-wb**

To capture the binding of Oct4 and Sox2 to form the complex (Oct4-Sox2) and the backwards reaction of unbinding, we modified the mathematical model (specifically the rate equations representing the dynamics of Oct4 ($X_{7}$), Sox2($X_{8}$), and Oct4-Sox2($X_{9}$). The changes were modified to identify whether the more simplistic model of RACIPE could be used or if a more complicated framework of RACIPE with binding/unbinding (RACIPE-wb) is needed. The equation for Oct4-Sox2 was changed to be entirely a binding/unbinding reaction instead of transcriptional activation and degradation. Since, the binding and unbinding of the Oct4-Sox2 complex also changes the concentration of Sox2 and Oct4 we included the binding and unbinding interactions into the rate of change equations for Sox2 and Oct4. The updated dynamics are given by the deterministic rate equations:

$$\dot{X_{1}}=\frac{G_{X_{1}}}{\lambda_{X_{1}X_{1}}^{+}*\lambda_{X_{7}X_{1}}^{+}}{*H}^{S}\left( X_{1}, X_{1}X_{10},n_{X_{1}X_{1}},\lambda_{X_{1}X_{1}}^{+} \right){*H}^{S}\left( X_{5}, X_{5}X_{10},n_{X_{5}X_{1}},\lambda_{X_{5}X_{1}}^{-} \right){*H}^{S}\left( X_{7}, X_{7}X_{10},n_{X_{7}X_{1}},\lambda_{X_{7}X_{1}}^{+} \right)*H^{S}\left( X_{8}, X_{8}X_{10},n_{X_{8}X_{1}},\lambda_{X_{8}X_{1}}^{-} \right)- K_{X_{1}}*X_{1}$$

$$\dot{X_{2}}=\frac{G_{X_{2}}}{\lambda_{X_{1}X_{2}}^{+}*\lambda_{X_{3}X_{2}}^{+}}{*H}^{S}\left( X_{1}, X_{1}X_{20},n_{X_{1}X_{2}},\lambda_{X_{1}X_{2}}^{+} \right){*H}^{S}\left( X_{3}, X_{3}X_{20},n_{X_{3}X_{2}},\lambda_{X_{3}X_{2}}^{+} \right)- K_{X_{2}}*X_{2}$$

$$\dot{X_{3}}=\frac{G_{X_{3}}}{\lambda_{X_{3}X_{3}}^{+}}{*H}^{S}\left( X_{3}, X_{3}X_{30},n_{X_{3}X_{3}},\lambda_{X_{3}X_{3}}^{+} \right){*H}^{S}\left( X_{5}, X_{5}X_{30},n_{X_{5}X_{3}},\lambda_{X_{5}X_{3}}^{-} \right){*H}^{S}\left( X_{7}, X_{7}X_{30},n_{X_{7}X_{3}},\lambda_{X_{7}X_{3}}^{-} \right)- K_{X_{3}}*X_{3}$$

$$\dot{X_{4}}=\frac{G_{X_{4}}}{\lambda_{X_{5}X_{4}}^{+}*\lambda_{X_{7}X_{4}}^{+}*\lambda_{X_{9}X_{4}}^{+}}{*H}^{S}\left( X_{5}, X_{5}X_{40},n_{X_{5}X_{4}},\lambda_{X_{5}X_{4}}^{+} \right){*H}^{S}\left( X_{7}, X_{7}X_{40},n_{X_{7}X_{4}},\lambda_{X_{7}X_{4}}^{+} \right){*H}^{S}\left( X_{9}, X_{9}X_{40},n_{X_{9}X_{4}},\lambda_{X_{9}X_{4}}^{+} \right)- K_{X_{4}}*X_{4}$$

$$\dot{X_{5}}=\frac{G_{X_{5}}}{\lambda_{X_{4}X_{5}}^{+}*\lambda_{X_{6}X_{5}}^{+}*\lambda_{X_{8}X_{5}}^{+}}{*H}^{S}\left( X_{1}, X_{1}X_{50},n_{X_{1}X_{5}},\lambda_{X_{1}X_{5}}^{-} \right){*H}^{S}\left( X_{3}, X_{3}X_{50},n_{X_{3}X_{5}},\lambda_{X_{3}X_{5}}^{-} \right){*H}^{S}\left( X_{4}, X_{4}X_{50},n_{X_{4}X_{5}},\lambda_{X_{4}X_{5}}^{+} \right)*H^{S}\left( X_{5}, X_{5}X_{50},n_{X_{5}X_{5}},\lambda_{X_{5}X_{5}}^{-} \right){*H}^{S}\left( X_{6}, X_{6}X_{50},n_{X_{6}X_{5}},\lambda_{X_{6}X_{5}}^{+} \right){*H}^{S}\left( X_{8}, X_{8}X_{50},n_{X_{8}X_{5}},\lambda_{X_{8}X_{5}}^{+} \right) - K_{X_{5}}*X_{5}$$

$$\dot{X_{6}}=\frac{G_{X_{6}}}{\lambda_{X_{5}X_{6}}^{+}}{*H}^{S}\left( X_{5}, X_{5}X_{60},n_{X_{5}X_{6}},\lambda_{X_{5}X_{6}}^{+} \right)- K_{X_{6}}*X_{6}$$

$$\dot{X_{7}}=\frac{G_{X_{7}}}{\lambda_{X_{8}X_{7}}^{+}}{*H}^{S}\left( X_{2}, X_{2}X_{70},n_{X_{2}X_{7}},\lambda_{X_{2}X_{7}}^{-} \right){*H}^{S}\left( X_{3}, X_{3}X_{70},n_{X_{3}X_{7}},\lambda_{X_{3}X_{7}}^{-} \right){*H}^{S}\left( X_{8}, X_{8}X_{70},n_{X_{8}X_{7}},\lambda_{X_{8}X_{7}}^{+} \right)- K_{X_{7}}*X_{7}-r_{binding}*X_{7}*X_{9}+r_{unbinding}*X_{8}$$

$$\dot{X_{8}}=r_{binding}*X_{7}*X_{9}- r_{unbinding}*X_{8}$$

$$\dot{X_{9}}=\frac{G_{X_{9}}}{\lambda_{X_{8}X_{9}}^{+}}{*H}^{S}\left( X_{8}, X_{8}X_{90},n_{X_{8}X_{9}},\lambda_{X_{8}X_{9}}^{+} \right)- K_{X_{9}}*X_{9}-r_{binding}*X_{7}*X_{9}+r_{unbinding}*X_{8}$$

The thresholds $X_{i}X_{j0}$ for a gene without regulation (aka a standalone gene [A]) are given by a randomization of the production and degradation rate. The [B] represents a gene whose dynamics are determined by a binding/unbinding rate equation the ‘standalone’ threshold is given by the equilibrium distribution. The [C] represents genes that have both transcription and binding/unbinding interactions the threshold is dependent on production and degradation rates, binding and unbinding rates, and the standalone distributions for a gene without regulation and a gene dependent on binding/unbinding. The equations are given by:


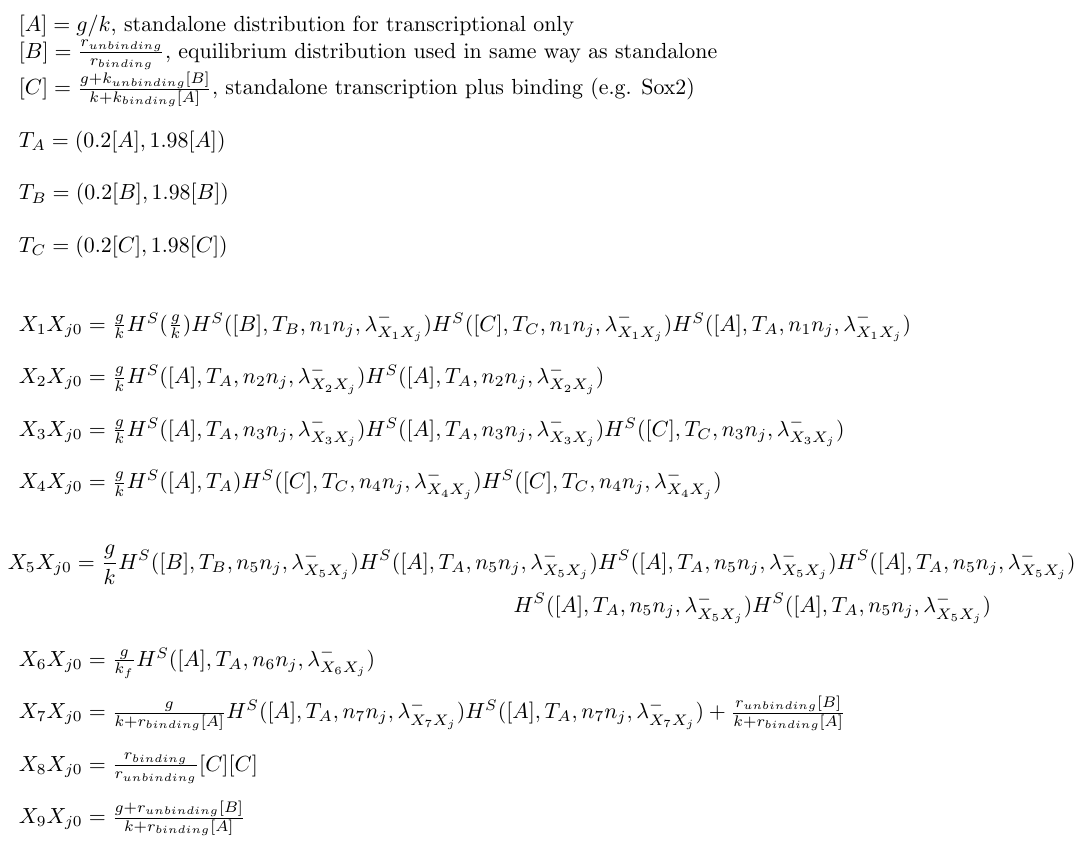


Where *r_binding_* is the rate of binding and *r_unbinding_* is the rate of unbinding and *r_equilibrium_ = r_unbinding_/ r_binding_* = [Oct4] [Sox2]/[Oct4-Sox2]. Therefore, the equilibrium rate is limited by either the amount of [Oct4] or [Sox2] resulting in [Oct4] ==[Oct4-Sox2] or [Sox2] ==[Oct4-Sox2]. Both of these result in *r_equilibrium_* being equal to the concentration of either [Oct4] or [SOX2] which we can estimate as a standalone gene distribution, similar to the threshold estimation in the first part of RACIPE. For our model, a standalone gene distribution is given by G_i_/k_i_. So *r_equilibrium_* should be near the range of [1,1000]. Since this was simply an estimation, we ran 24 simulations of 1000 parameters solving the ODEs using 100 different initial conditions. The 24 simulations were the same except for the values of *r_binding_* and *r_unbinding_*. Based on these results the range for *r_equilibrium_* can be as small as [1,100] and as large as [0.01,1000] without significant differences in the results. The range of [0.1,100] was taken as a sufficient range and is used in the main text for results modeled using the RACIPE with binding (RACIPE-wb) framework (labeled as par3 or p3 in SI). Additional ranges of [0.01-100] (labeled as par1 or p1) and [0.1-10] (labeled as par2 or p2) are compared in **Table S2-S3** and **figures S17-S21).** The gene expression data was normalized self-consistently as previously mentioned. The data was also compared to the results from the original RACIPE framework and was normalized by using the z-scores from the original RACIPE results. All data is assumed to be self-consistently normalized unless otherwise stated. The estimated thresholds for each gene using RACIPE-wb are compared to the thresholds from original RACIPE in **Table S3** showing no significant change for any genes except Sox2, Oct4, and the OCT4-SOX2 complex. This suggests that the binding is only affecting the genes that we would expect. Additionally, **figure S17** shows that all RACIPE frameworks satisfy the half-functional rule. By comparing the equilibrium state found with the model to the expected result (**figure S18)** also shows that the RACIPE-wb framework satisfies the half-functional rule. **Figure S19** shows that results for principal component analysis when the results for RACIPE-wb are both self-normalized and normalized via the z-scores of the original data. The results show that the original RACIPE framework predicts similar clusters but are not as distinct as when binding/unbinding is included. The different gene states (colored parts of the tree) were found by using hierarchical clustering analysis (**figure S20**). The clustering results show an overall agreement between the original RACIPE framework and RACIPE-wb. Additionally, the gene states were mapped onto the principal components (**figure S21**) which confirms that the two main clusters in the PCA results are equivalent to the two main gene states found using unsupervised HCA.

1. **Normalization of gene expression data**

From an ensemble of the RACIPE models, we collected a large set of gene expression data, analogous to those obtained in experiment. Therefore, we can apply tools for data analysis to these computationally generated data. We processed RACIPE-generated gene expression data by a standard normalization method before we performed further analysis.

The gene expression levels from the WT models are normalized by first log transformation and standardization, i.e.

$x_{i}^{WT}\to\frac{\log_{2}(x_{i}^{WT})-\overline{\log_{2}(x_{i}^{WT})}}{\sigma(\log_{2}(x_{i}^{WT}))}$, (2)

The gene expression levels from the treatment models is normalized by first log transformation and standardization by the mean ($\overline{\log_{2}(x_{i}^{WT})}$) and the standard deviation ($\sigma(\log_{2}(x_{i}^{WT}))$) of the WT models

$x_{i}^{T}\to\frac{\log_{2}(x_{i}^{T})-\overline{\log_{2}(x_{i}^{WT})}}{\sigma(\log_{2}(x_{i}^{WT}))}$, (3)

For the single cell gene expression data of mouse embryo cells [[30]](https://paperpile.com/c/rr7uYp/GcBN), we followed the procedures given in the original paper for data normalization.

1. **Clustering analysis**

We performed clustering analysis on the normalized gene expression data from the RACIPE models as shown in **Fig. 3a** (left). Each column represents a gene, and each row represents a stable steady state of the circuit for a particular RACIPE model. For RACIPE models with more than one stable state, the gene expression profiles for all of the stable states were shown in multiple rows. We applied the average linkage hierarchical clustering analysis (HCA) using Euclidean distance to analyze both the RACIPE-generated gene expression data and the single-cell gene expression data of mouse embryo cells utilizing Cluster 3.0. The clustering results were visualized by JavaTreeview. The cutoff distance was chosen to be 0.8 so that major clusters have probability more than 0.5% and each cluster has a distinct gene expression pattern.

These clusters correspond to different gene states for the circuit. Principal component analysis (PCA) was performed using the “pca” function in Matlab/2014b. The major gene clusters can be readily recognizable from the probability density map projected onto the first two principal component axes (PC1 and PC2).

The single cell gene expression data of mouse embryo cells were analyzed by the same average linkage hierarchical clustering analysis with Euclidean distance, from which we identified the same nine clusters (**Fig. S6**) as those found in the original paper. To compare the experimental data with the predicted data from the RACIPE, we extracted the data of the 6 common genes (Gata6, Cdx2, Klf4, Nanog, Oct4, and Sox2), as shown in the second heat map (**Fig. 3a**, right).

1. **Random gene expression data**

Throughout the study, we utilized randomly generated gene expression data for statistically significant tests. We first computed the histogram of gene expression for each gene from all the RACIPE models. A random gene expression vector was constructed so that each element has a value randomly sampled from the histogram of the corresponding gene.

1. **Comparison between the gene expression data from the RACIPE models and the experiments**

The similarity between two gene expression vectors (X and Y) can be quantified by the mean square error ($MSE^{XY}$)

$MSE^{XY}=\frac{1}{n}\left\|X-Y\right\|_{2}^{2}$ (4)

where X and Y are the gene expression vectors with the same number of genes n. The choice of X and Y will be explained in the next two paragraphs. X and Y were regarded to be significantly similar if $MSE^{XY}$ is better than 99% $MSE^{XZ}$, where

$MSE_{i}^{XZ}=\frac{1}{n}\left\|X-Z_{i}\right\|_{2}^{2}$, (5)

Z consists of 10,000 random gene expression vectors (each vector is labeled by i, see the above section).

In our cluster-based analysis, an experimental gene state cluster was matched to a gene state cluster from RACIPE as follows. We calculated the $MSE^{XY}$ between the average gene expression vector of the experimental cluster (X) and the average gene expression vector for each RACIPE cluster (Y) (**Fig. S7**). Then, we calculated the $MSE^{XZ}$ between the average gene expression vector of the experimental cluster (X) and each of the 10,000 random gene expression vectors (Z). The experimental cluster was considered to be significantly similar to a RACIPE cluster when the $MSE^{XY}$ value is smaller than 99% of the $MSE^{XZ}$ values.

In our individual-based analysis, we calculated the fraction of the RACIPE models whose gene expression vectors match any one of the experimental clusters by a one-on-one comparison. For each experimental gene state cluster, we calculated the $MSE^{XY}$ between the average gene expression vector of the experimental cluster (X) and the gene expression vector of each stable state for every RACIPE model (Y). The RACIPE model was considered to be significantly similar to one of the experimental clusters according to the above criterion.

We also generated null models to derive p values for the comparison between the experimental data and the RACIPE data. For the cluster-based analysis, we generated 15 random gene expression vectors to represent the average gene expression vectors of 15 gene states, and randomly assigned the probability of a different RACIPE cluster to each vector. This data serves as a null model, and they were compared with the experimental data. This procedure is repeated for 10,000 times for statistical analysis. For the individual-based analysis, we performed two random tests. First, we generated the same number of random gene expression vectors as the RACIPE models and used them as the null model. Second, the null model is the same RACIPE models, but for each cluster the gene identities were shuffled.

1. **Comparison of the parameters between two gene states.**

Each parameter from the RACIPE model ensemble was first standardized by subtracting its mean and dividing by its standard deviation. Then, we found the RACIPE models (group I) that have stable states from the gene state cluster i but no states from the cluster j. Similarly, we searched for the RACIPE models (group J) that have stable states from the gene state cluster j but no states from the cluster i. The mean of each parameter in both groups was plotted in a 2D diagram (**figure 5**). Note that the fold changes of the inhibitory regulation were inverted before the normalization.

We applied the RACIPE to each network with randomized connections (figure S9). Using the same cutoff distance as the stemness GRN, we identified major clusters (gene states) by using hierarchical clustering analysis (**figure S12**). The clusters from the random circuits were compared with experimental data by both the cluster-based and the individual-based methods (**figure S13**).

We used k nearest neighbors approach to estimate the local density around each data point in the high dimensional gene expression space. The local density of a point x is

$P(x)\cong\frac{k}{NV}=\frac{k}{N\cdot c_{D}\cdot R_{k}^{D}(x)}$, (6)

where k is number of nearest neighbors for the density estimation (set to be 100 here), N is the total number of data points and V is the volume that encloses the k neighbors surrounding the point x. $c_{D}$ is the volume of the unit sphere in D dimensions, which is equal to $c_{D}=\pi^{D/2}/\Gamma(D/2+1)$. $R_{k}^{D}(x)$ is the distance between the point x and its k-th nearest neighbor.

1. **Classification of the gene expression data**

By the hierarchical clustering analysis of the RACIPE models, we identified 15 major gene state clusters (**figure 3a**). The gene expression data of these gene states serve as the references for later classification of new gene expression data. To do this, we assigned the unclassified gene expression profile (i) to the gene state cluster with the smallest “minimum Euclidean distance” ($D_{in}$) between the i and the reference gene state n. $D_{in}$ is defined as

, (7)

where $N_{n}$ is the number of gene expression data in the gene state cluster n (n = 1, 2 … 15). $X_{ig}$ is the expression level of gene g in the steady state i, and $R_{njg}$ is the expression level of gene g for every steady state j in the reference gene state n.

To calculate the probability of observing each gene state cluster from all the RACIPE models (**figures 2b, S1**), we computed the weighted count of all the gene expression data that belong to the cluster. We weighted each gene expression data by the number of stable steady states for the corresponding RACIPE model. For example, if the RACIPE model is bi-stable, the contributions of both of the stable steady state data are ½ instead of 1.

1. **Perturbation treatment**

For the perturbation treatment, we generated a new ensemble of models by specifying certain parameters and randomizing the rest parameters in the same ranges as before. We simulated the knockout (KO) of a gene by setting the maximum production rate ($G_{i}$) of the gene to be zero. For the linkage removal, we set the fold change of the regulation to be 1 (no fold change). For the activation (inhibition) of a gene, we increase the maximum production rates ($G_{i}$) by 50 times. We referred to the RACIPE models for the original circuit as wide-type (WT) models, and those for the circuit under certain treatment as treatment (T) models.

1. **Analysis of knockout**

To measure the effects of the gene knockout, we calculated Kullback–Leibler (KL) divergence (D_kl_) for the knockout of each gene, defined as

$D_{KLi}(P_{WT}||P_{iKO})=\sum_{n} P_{WT}(n)\ln\frac{P_{WT}(n)}{P_{iKO}(n)}$, (8)

where $P_{WT}$ is the probability distribution of the number of stable steady states for each RACIPE model for the circuit without treatment (WT), and $P_{iKD}$is that for the circuit with the knockout (KO) of gene i. n stands for the RACIPE models with n stable states. The KL divergence was also applied to evaluate the effects of the removal of each regulatory link.

1. **Population heterogeneity**

We quantified the population heterogeneity by calculating the weighted information entropy (phylogenetic)(56)

$H=-\sum_{branches b of T} l(b)P(b)\log_{2}P(b)$, (9)

where T is a tree derived from the hierarchical clustering analysis on the RACIPE models (Fig. 3a, left), $l(b)$ is the length of a branch b of the T, and $P(b)$ represents the probability of all the gene states that are represented by the leaves descending from b. More heterogeneous population tends to have higher entropy.

**Supplementary Table**

**Table S1** **Ranges for parametric randomization in RACIPE**

| Parameters | Range |
| --- | --- |
| Maximal production rate () | 1-100 |
| Degradation rate () | 0.1-1 |
| Fold change ()* | 1-100 |
| Threshold ($X_{0}$) | 0.7-69.3 |
| Cooperativity of the regulation () | 1, 2, 3, 4, 5, 6 |

* For inhibition, fold change ranges from 0.01 to 1, but a uniform distribution is sampled for and then the inverted value is chosen at the fold change due to that inhibitory link.

**Table S2** **Ranges for parametric randomization in RACIPE-wb**

| Parameters | Set | Range |
| --- | --- | --- |
| r_binding_ | par1 | 0.001-0.1 |
|  | par2 | 0.01-0.1 |
|  | par3* | 0.001-0.1 |
| r_unbinding_ | par1 | 0.001-0.1 |
|  | par2 | 0.01-0.1 |
|  | par3* | 0.1-1.0 |
| Maximal production rate () | par1, par2, par3* | 1-100 |
| Degradation rate () | par1, par2, par3* | 0.1-1 |
| Fold change () | par1, par2, par3* | 1-100 |
| Threshold (X_0_) | par1, par2, par3* | See Table S3 |
| Cooperativity of the regulation () | par1, par2, par3* | 1,2,3,4,5,6 |

The ranges for all other parameters are the same as for the original RACIPE framework. * Ranges for par3 are used in the main text for results labeled RACIPE-wb.

**Table S3: The estimated thresholds by RACIPE and RACIPE-wb**

|  | Gcnf | Cdx2 | Gata6 | Pbx1 | Klf4 | Nanog | Oct4 | Oct4-Sox2 | Sox2 |
| --- | --- | --- | --- | --- | --- | --- | --- | --- | --- |
| RACIPE | 12.4 | 2.39 | 0.83 | 38.13 | 3.95 | 0.08 | 2.39 | 12.4 | 38.13 |
| RACIPE-wb (par1, r_eq_ = [0.01,100]) | 12.415 | 2.075 | 0.726 | 37.262 | 3.535 | 0.08 | 0.323 | 0.991 | 3.921 |
| RACIPE-wb (par2, r_eq_ = [0.1,10]) | 13.795 | 2.363 | 0.856 | 41.402 | 3.944 | 0.095 | 0.421 | 1.905 | 3.995 |
| RACIPE-wb (par3, r_eq_ = [0.1,100]) | 13.782 | 2.302 | 0.777 | 41.36 | 3.933 | 0.096 | 0.533 | 2.074 | 4.590 |

The first row contains the estimated threshold values for each TF in the shifted Hill function by RACIPE. The bottom three rows contain the estimated threshold values for each TF by RACIPE-wb for 3 scenarios characterized by different ranges of $k_{eq}$. The table shows that only the estimated thresholds for Oct4, Sox2, and the complex OCT4-SOX2 differ greatly by RACIPE-wb relative to those by RACIPE.

**Supplementary Figures**

a

**
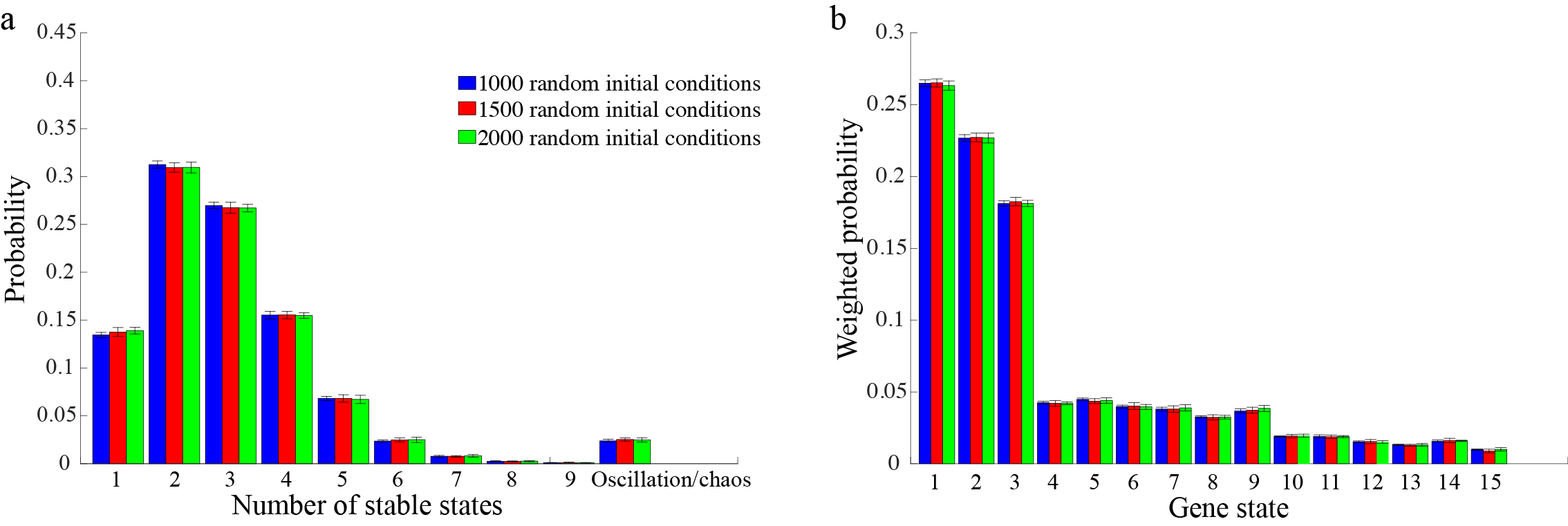
**

b

**
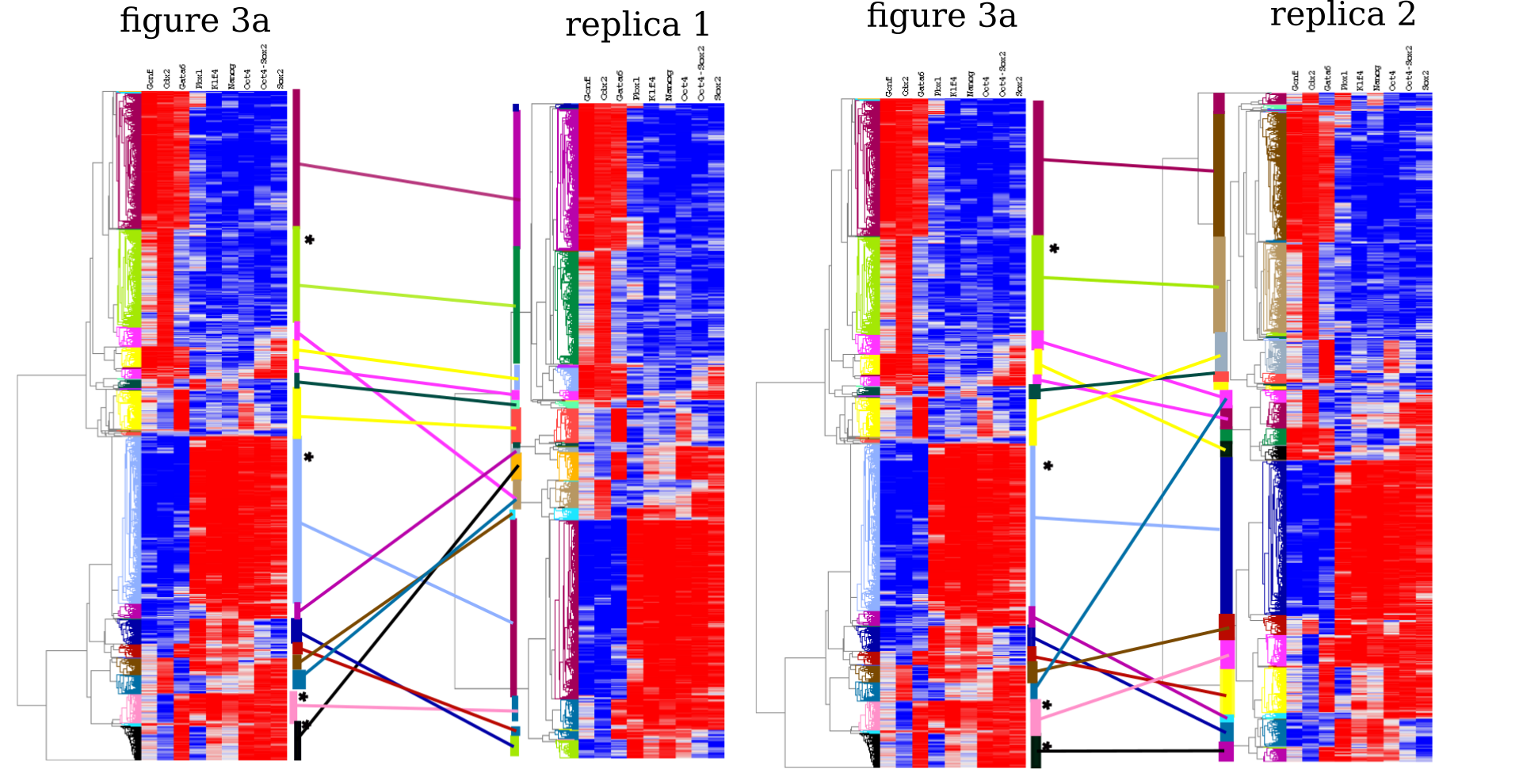
**

c

**
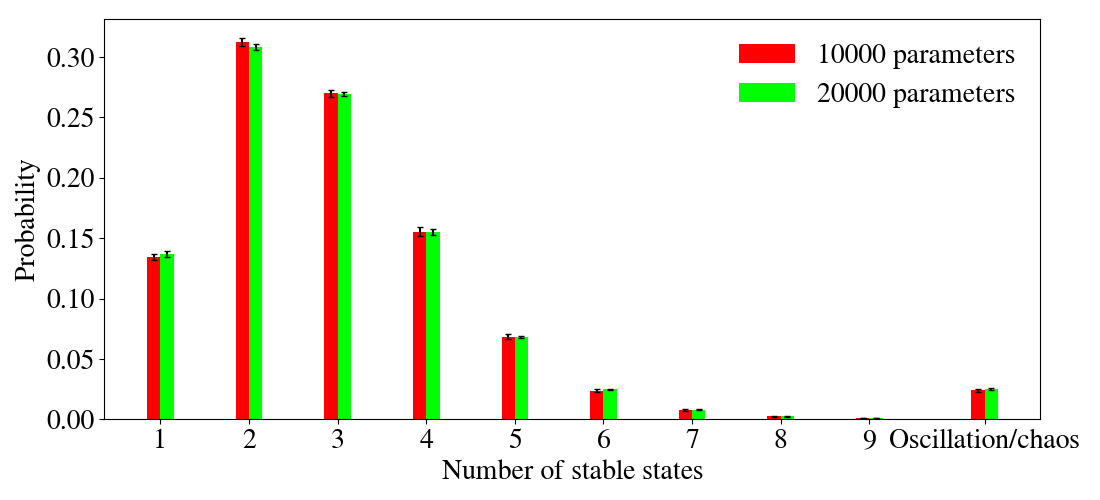
**

**Figure S1.** **The effects of the number of random initial conditions and parameter sets on the convergence of the gene states.** In (a), for each RACIPE model, all the possible stable steady states are obtained by running ODE simulations starting from different random initial conditions. Here, we checked whether the statistical results converged by varying different numbers of initial conditions (blue: 1000 times, red: 1500 times, and green: 2000 times). Each case was repeated 10 times to estimate the mean and the standard deviation of the histogram. (b) Comparison of the HCA results between RACIPE-generated gene expression profiles using 10,000 sets of parameters (as shown in **figure 3a**) and gene expression profiles using another 2 distinct 10,000 sets of parameters, referred to as replica 1 and replica 2 respectively. Each column represents a gene and each row represents a stable steady state. Comparison results show that 14 gene states shown in figure 3a are repeatedly observed in both replicas. The lines connect the same gene state from distinct 10,000 sets of parameters. The star “*” highlights the four gene states that recapitulate the single-cell gene expression data of mouse ESCs at 32-cell and 64-cell stages. The result suggests 10,000 parameters are indeed sufficient to capture the robust gene states. (C) The probability distribution of the number of stable states for the RACIPE models for different numbers of parameter sets (red: 10,000 sets and green: 20,000) used to solve the rate equations. Each was computed for 10 different sets of initial conditions to estimate the mean and standard deviations for the histogram. There is no significant difference between the results for any number of stable states.


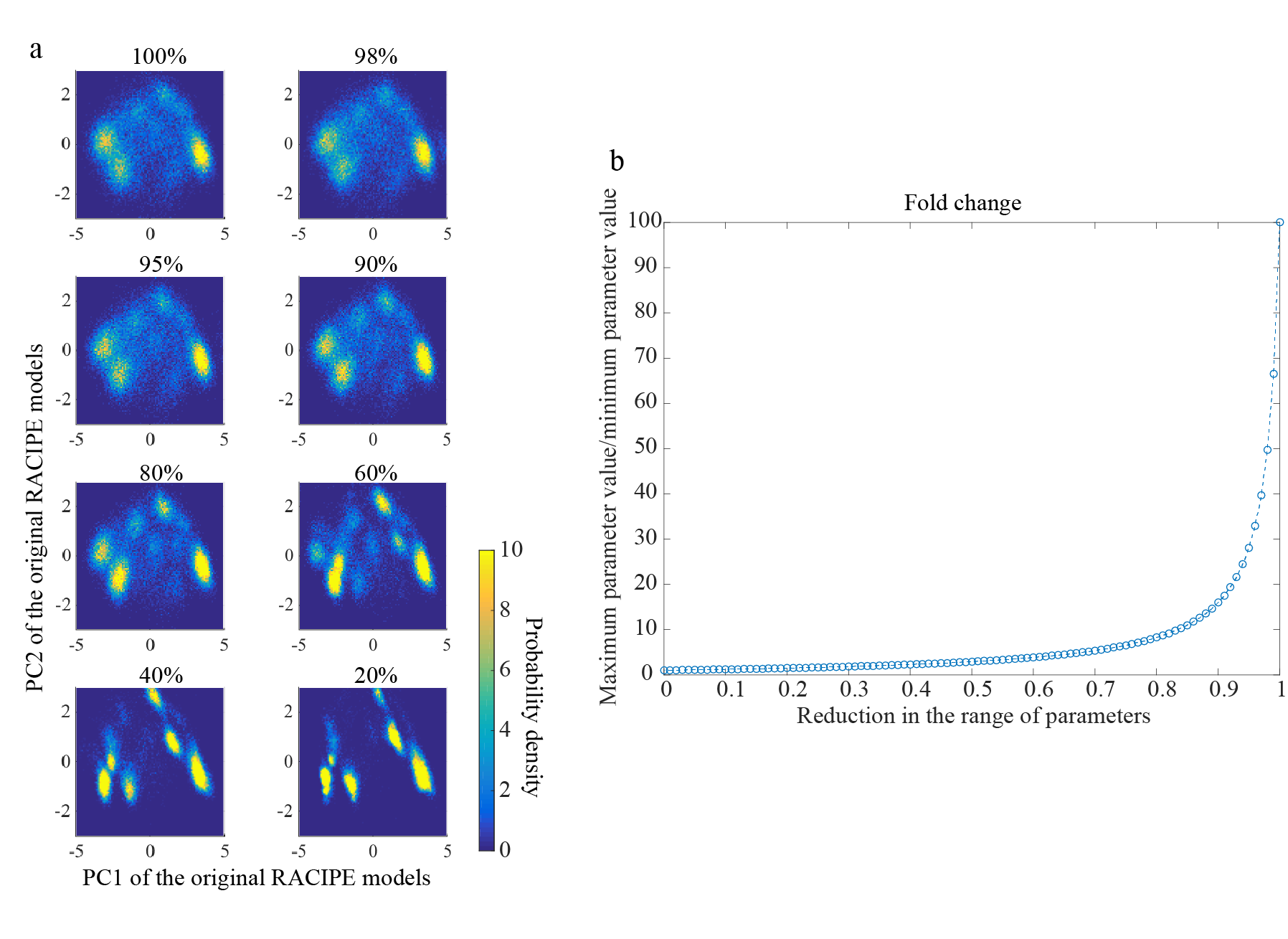


**Figure S2. Dependence of the RACIPE gene expression data on the range of the parameters for randomization.** (a) 2D probability density maps of the RACIPE gene expression data projected to the 1st and 2nd principal component axes of the original RACIPE models (as shown in figure 2c). For each small panel, the parameters were randomly selected from different ranges – the original RACIPE protocol was labeled as “100%”; for the other cases, the ranges are reduced to the corresponding percentages. The locations of the major clusters in these plots are similar till the ranges are reduced to 60%. (b) The ratio of the maximum and minimum value of fold change (λ) parameters as the function of the shrink of the original range of the parameters for randomization. When there is no reduction (1.0 in the x-axis), the ratio is 100. But the ratio dramatically reduces to about only 10 when the range is reduced to 80%.


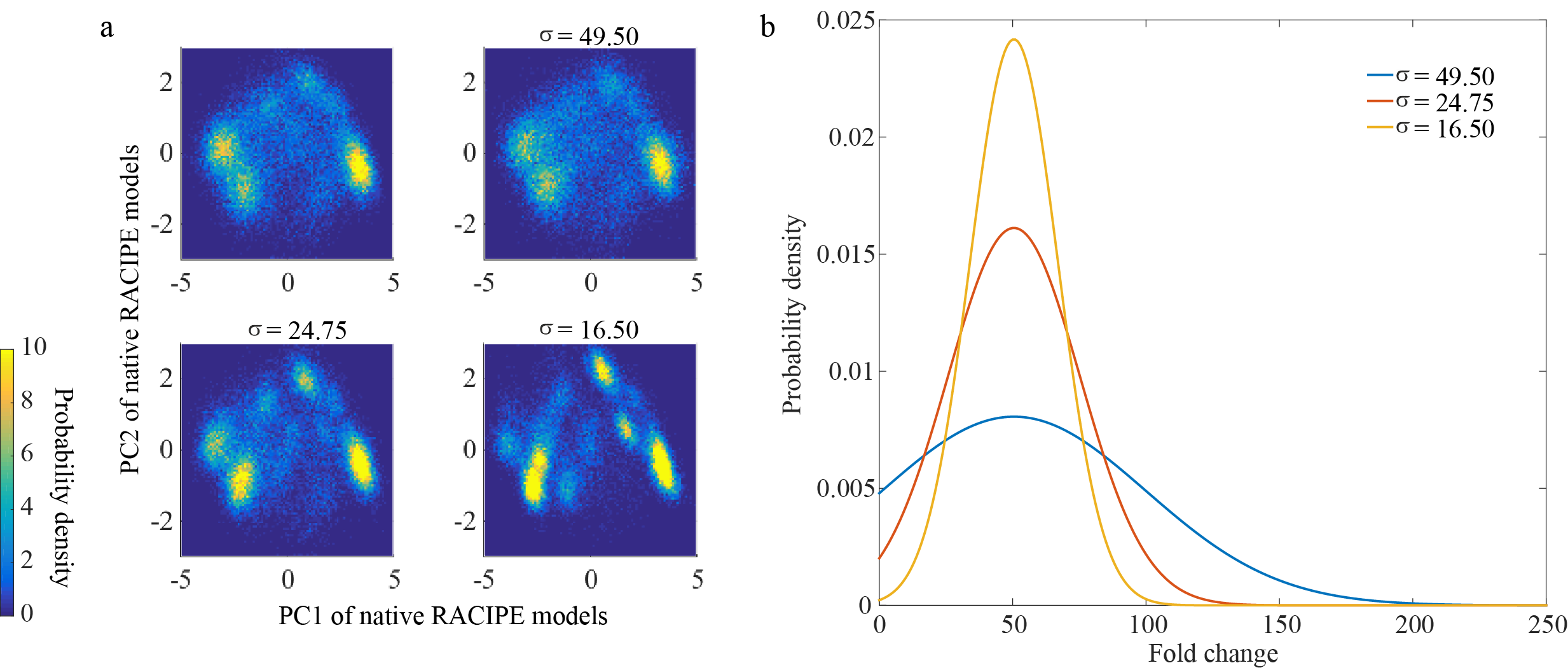


**Figure S3. Dependence of the RACIPE gene expression data on the type of the sampling distribution.** So far, we only tested the RACIPE protocol in which the parameters are randomly sampled from uniform distributions. Here we tested RACIPE using Gaussian distribution. (a) 2D probability density maps of the RACIPE gene expression data projected to the 1st and 2nd principal component axes of the native RACIPE models (as shown in **figure 2c**). Top left panel: the standard RACIPE method; the rest panels: the RACIPE protocols using different Gaussian distributions (standard deviations of fold change parameter for each case are shown on the top). The locations of the major clusters in these plots are similar except for the one at bottom right. (b) The Gaussian distribution of the fold change (λ) parameters for each case: the blue curve corresponds to the top right panel; the standard deviations of the Gaussian distribution were taken as half of the standard deviations of the uniform distribution. The red curve corresponds to the bottom left panel, and the orange curve corresponds to the bottom right panel.


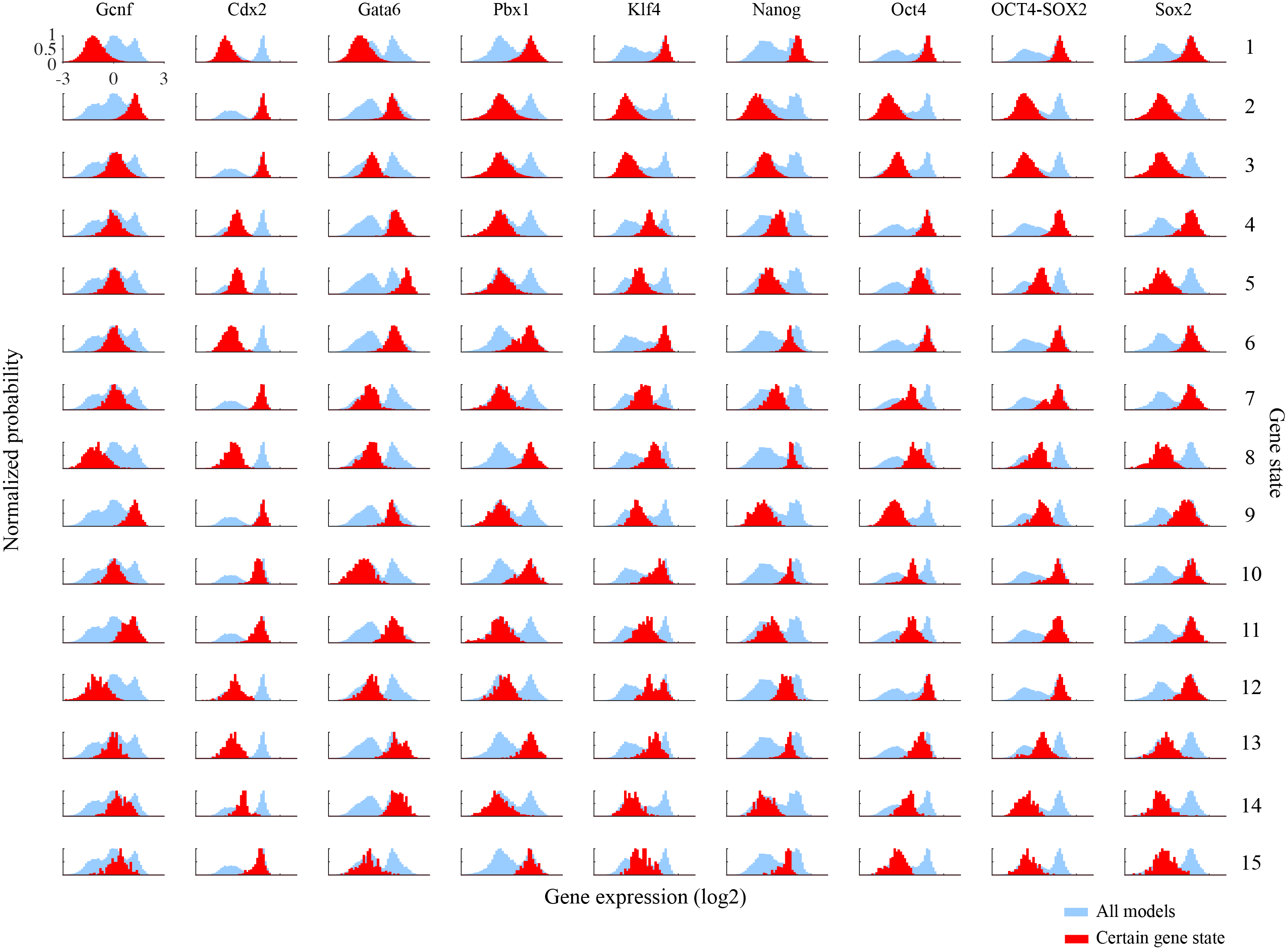


**Figure S4. Gene expression distributions of each gene state.** The gene expression distribution of each gene for all of the RACIPE models is shown in blue, while that for each gene state is shown in red (50 bins for the histogram of each distribution). For clarity, each distribution is normalized by its maximum probability. Each column represents a gene and each row represents a gene state. The results illustrate that RACIPE, unlike the traditional Boolean network model (1), allows the identification of the intermediate expression for some gene states.

**
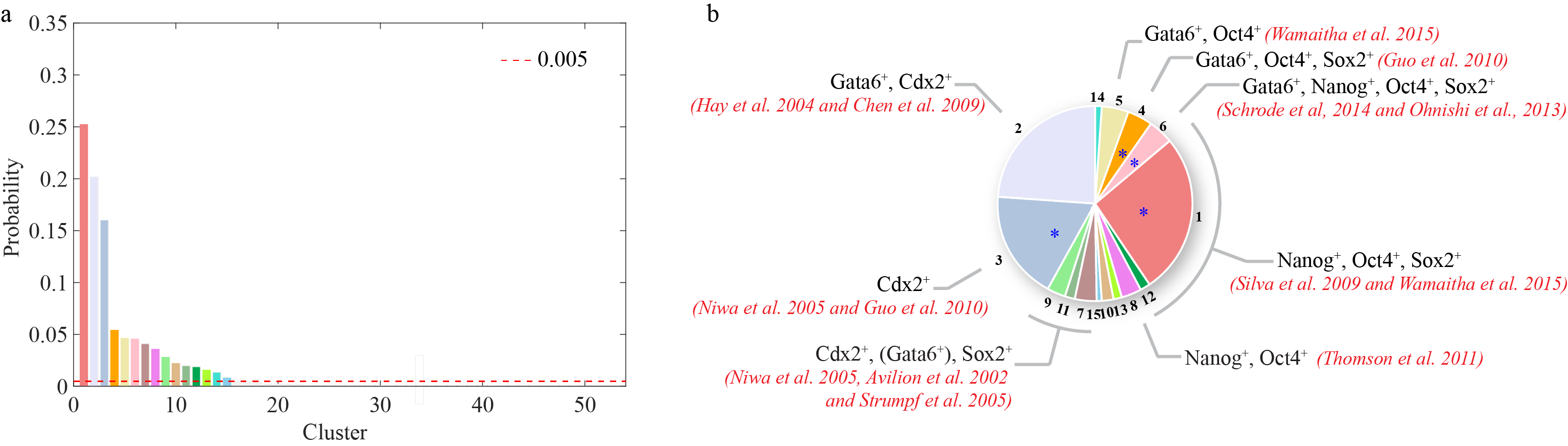
**

**Figure S5.** Characteristic gene expressions and experimental evidences for each of these gene states, ranked by the likelihood in the RACIPE models as in **figure 3b**. The four gene states that match the single cell experimental data (**figure 3a**) are highlighted by blue asterisks.


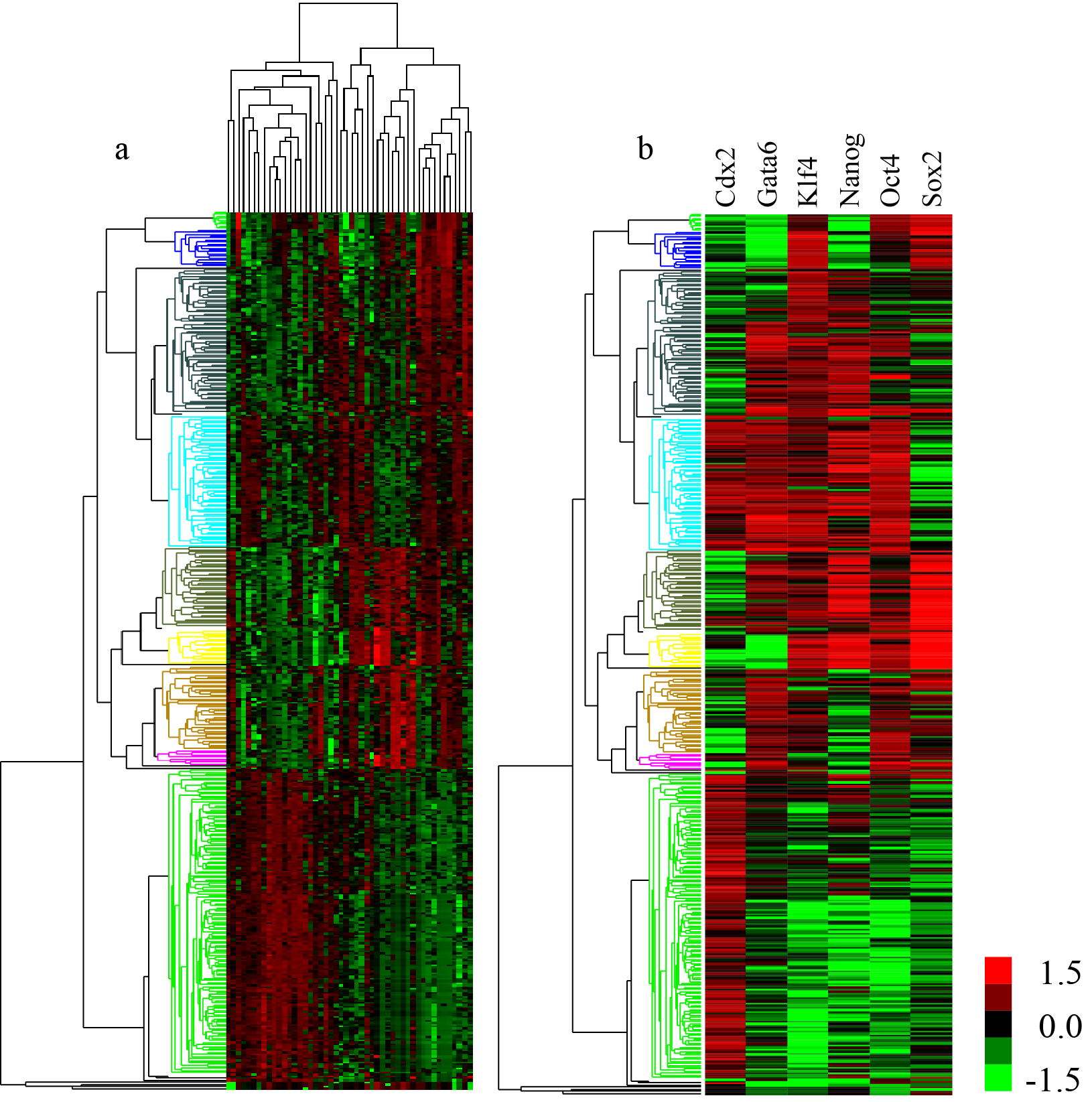


**Figure S6.** **Hierarchical clustering of mouse embryo single cell gene expression data**. (a) Clustering result of a total of 48 genes (2). (b) The extracted gene expression data for 6 genes (Cdx2, Gata6, Klf4, Nanog, Oct4 and Sox2). Each column represents a gene while each row represents a cell.


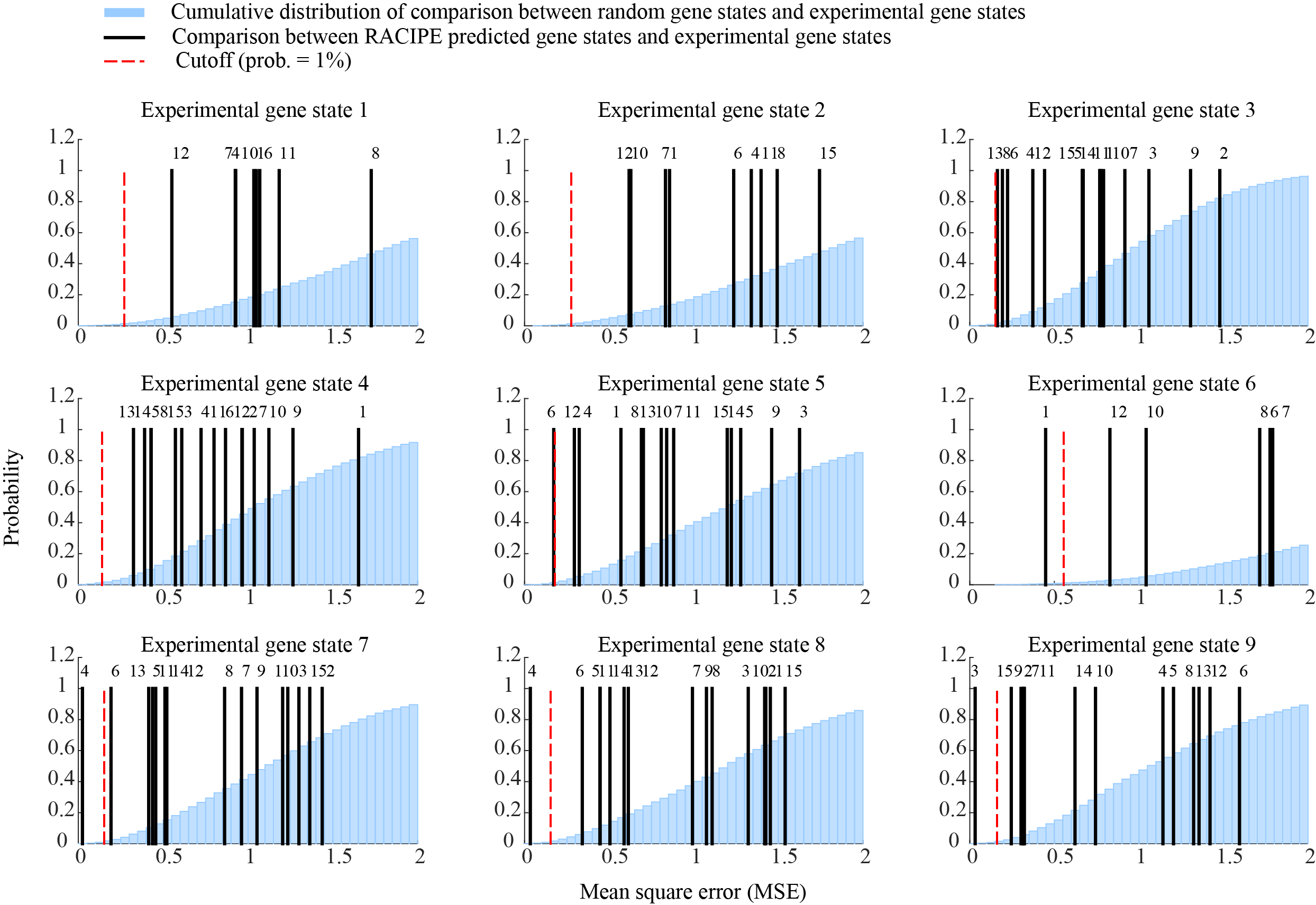


**Figure S7. Comparison between the experimental gene states and the RACIPE gene states.** The comparison analysis was done by the cluster-based analysis (See Methods). First, we calculated the average gene expression vector of each experimental cluster (experimental gene state) and each RACIPE cluster (RACIPE gene state). Then, for each experimental gene state, we calculated the mean square errors (MSEs, see Methods) between the average gene expression vector of the experimental gene state and that of every RACIPE gene state (shown as solid lines, labeled on the top). The blue histogram is the cumulative probability distribution of the MSEs between the average gene expression vector of the experimental gene state and 10,000 random gene expression vectors. We considered that an RACIPE gene state matches the experimental gene state if the corresponding MSE between them is below 1% of the cumulative probability (the cutoff, shown as red dashed lines). According to the plots, no match was found for the 1^st^, 2^nd^, 3^rd^ and 4^th^ experimental gene states. The 5^th^ experimental gene state matches the 6^th^ RACIPE gene state; the 6^th^ experimental gene state matches the 1^st^ RACIPE gene state; both the 7^th^ and the 8^th^ experimental gene states match the same 4^th^ RACIPE gene state; the 9^th^ experimental gene state matches the 3^st^ RACIPE gene state.

**Figure S8. Comparison between experimental gene expression data and random gene expression data.** Random resampling was performed to generate random datasets to calculate the statistical significance (i.e. p-value) of the comparison between experimental data and the RACIPE generated data. Here three different tests were performed. (a) Histogram of the number of experimental gene states identified by a random dataset. The RACIPE (red dotted line) outperforms more than 99% of random models. (b) Histogram of the fraction of a random dataset that matches the experimental gene states by the cluster-based analysis. In this test, we generated 10,000 random datasets, each of which has 15 resampled gene expression vectors representing the average gene expression vectors of a null model. For each random vector, we also randomly assigned the probability of a different RACIPE cluster to the vector. (c) Histogram of the fraction of a random dataset that matches the experimental gene states by the individual-based analysis. Here we generated 10,000 random gene expression vectors (same size as the RACIPE dataset) resampled from the distributions of gene expression of each gene in the RACIPE dataset. (d) Similar to the individual-based analysis used in (c), but the random datasets were generated in a different way. Here, a random dataset was generated by shuffling the gene identities for each gene state cluster in the RACIPE dataset. For all the tests, we found the RACIPE dataset (red dotted line) outperforms almost all the random datasets (>99%, grey histogram) in matching the experimental dataset.


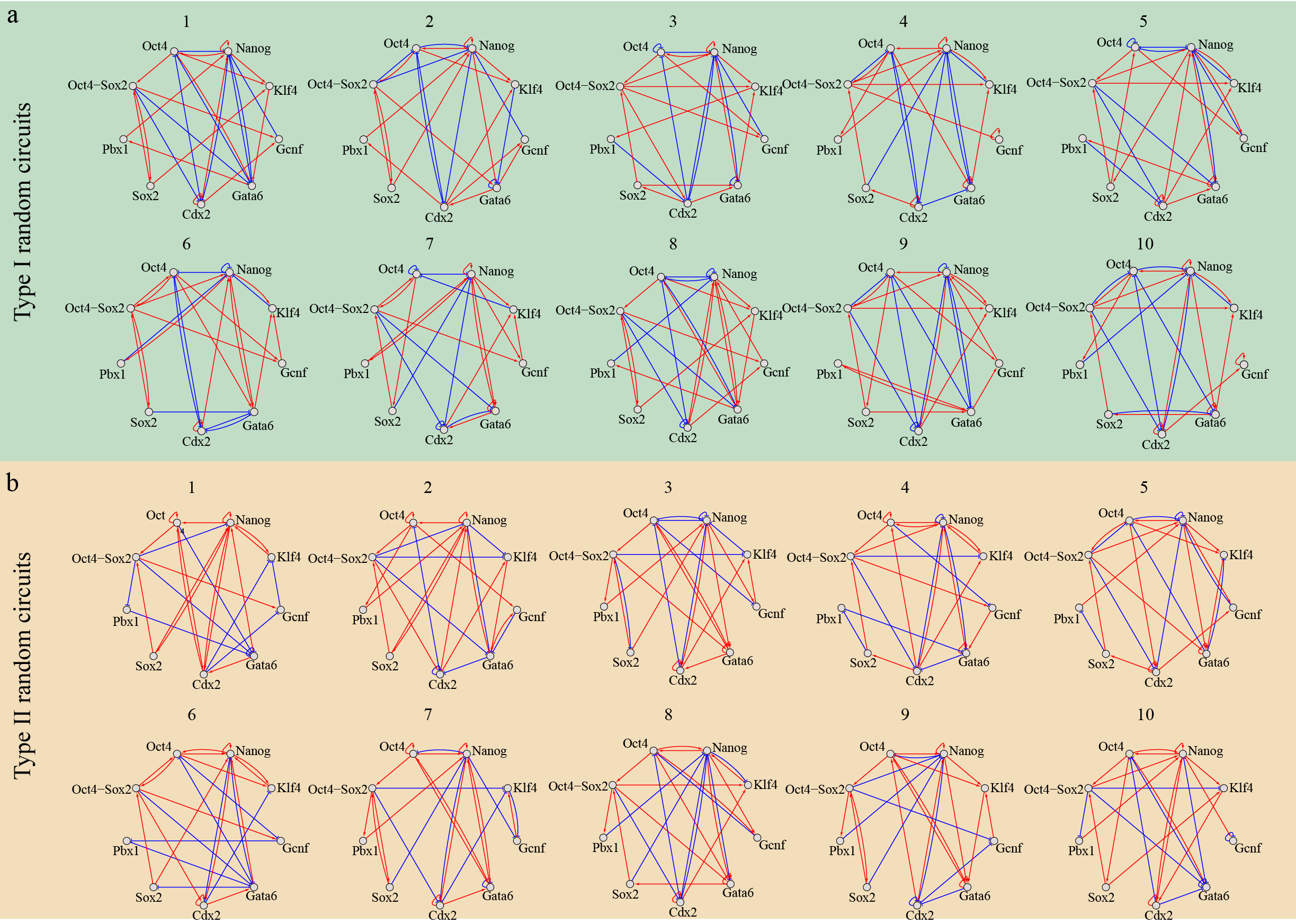


**Figure S9. Schematics of random circuits.** (a) Type I random circuits. (b) Type II random circuits. Red arrow lines stand for activations and blue bar lines stand for inhibitions. The index for each circuit is shown at the top.

**
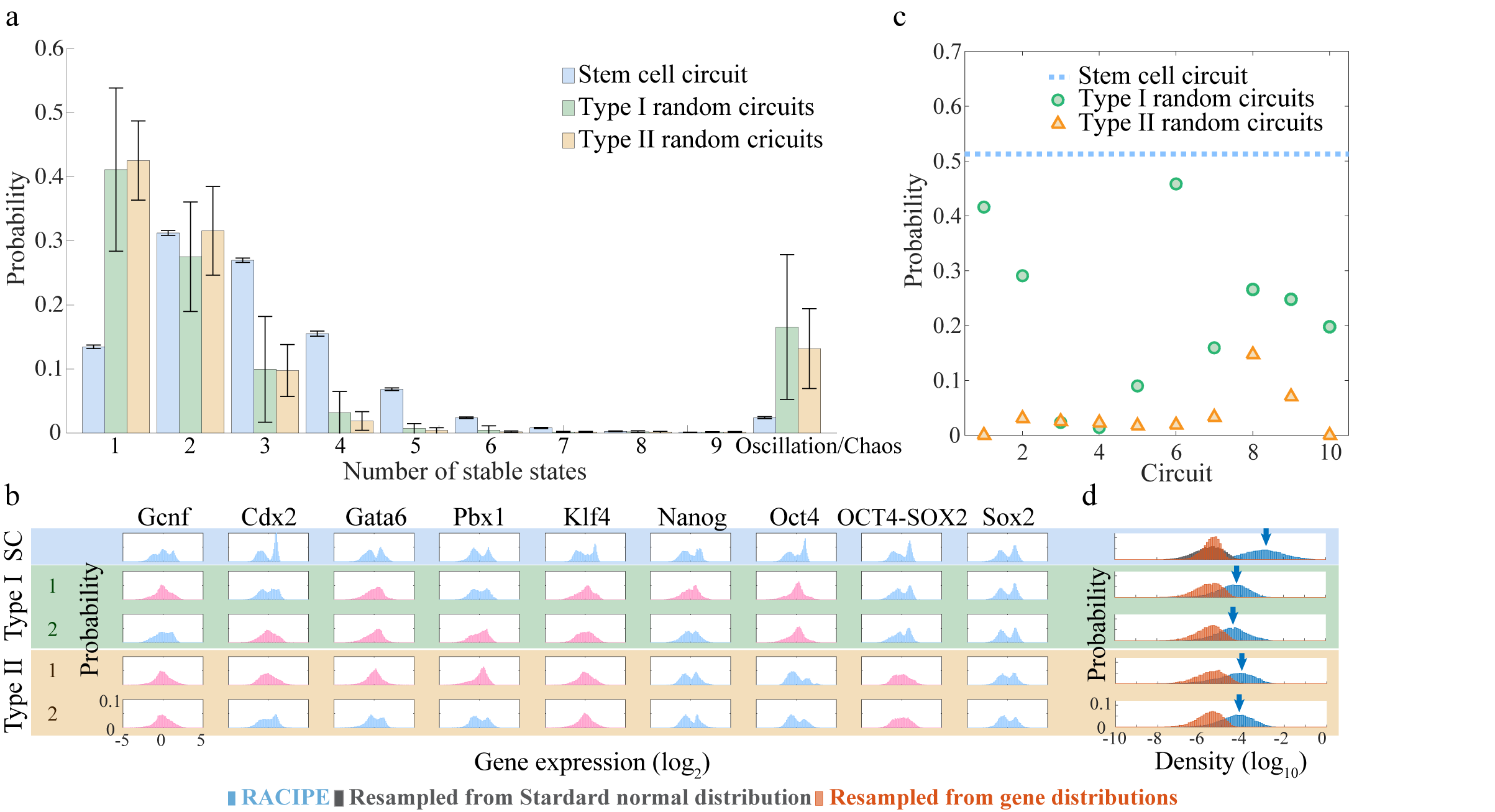
**

**Figure S10.** Probability distribution of the number of stable steady states of each RACIPE model for the stemness GRN and the randomized networks (type I: **figure S9a**, type II: **figure S9b**). The error bar for the stem cell circuit shows the standard deviation for 10 simulation repeats, while the ones for the random circuits represent the standard deviations for the 10 random circuits of each type.


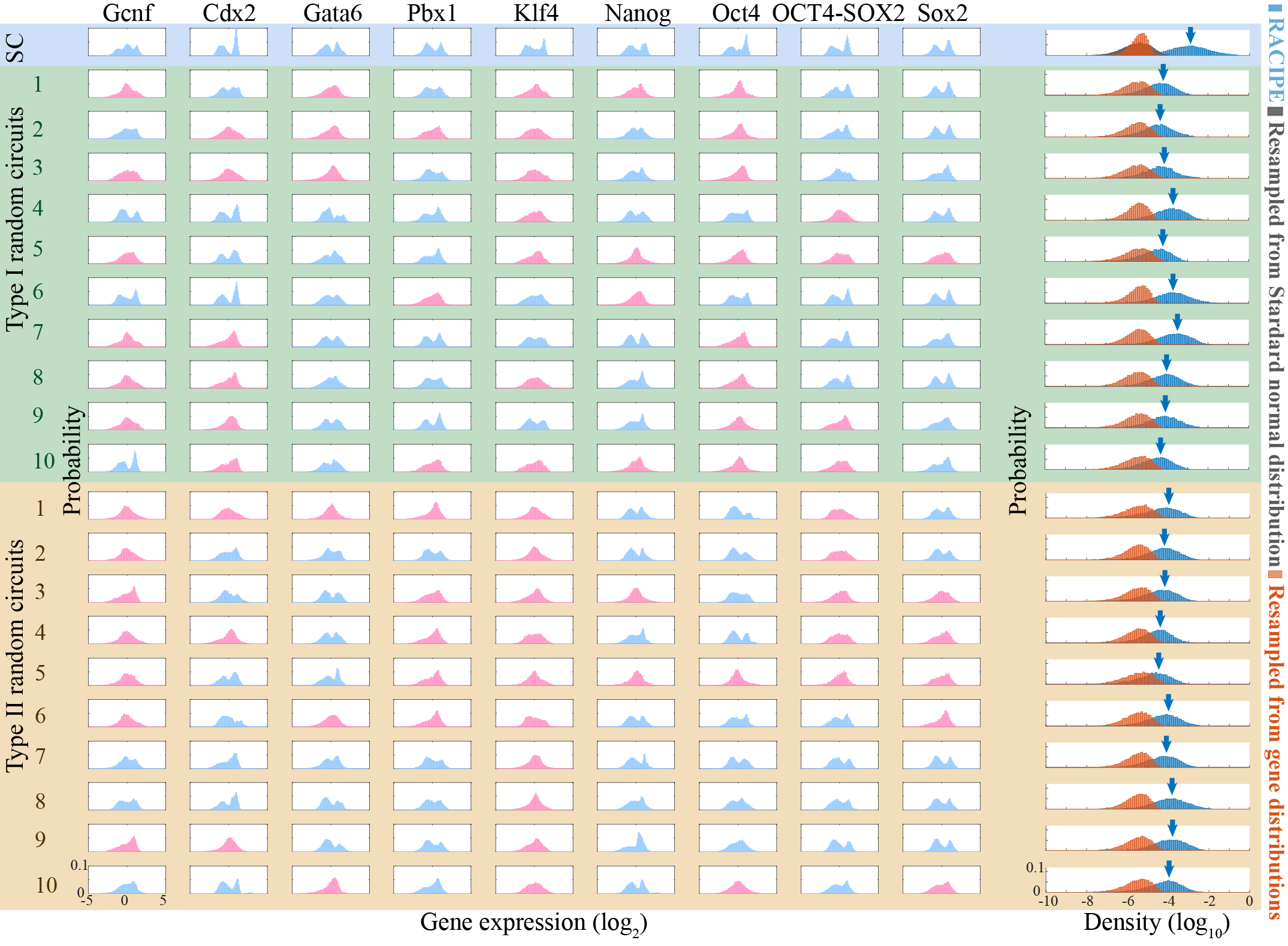


**Figure S11. Comparison between the stemness GRN and randomized stemness GRNs.** The figure is similar to **figure 3a**, but it includes the results for all of the random circuits. The columns present genes and rows correspond to different circuits. For the results of random circuits, the indices are consistent with those for the random circuits (**figure S9**). The distributions with only a single peak are highlighted by the pink color. The details to calculate the densities can be found in the **supplementary material**, **§S7.**


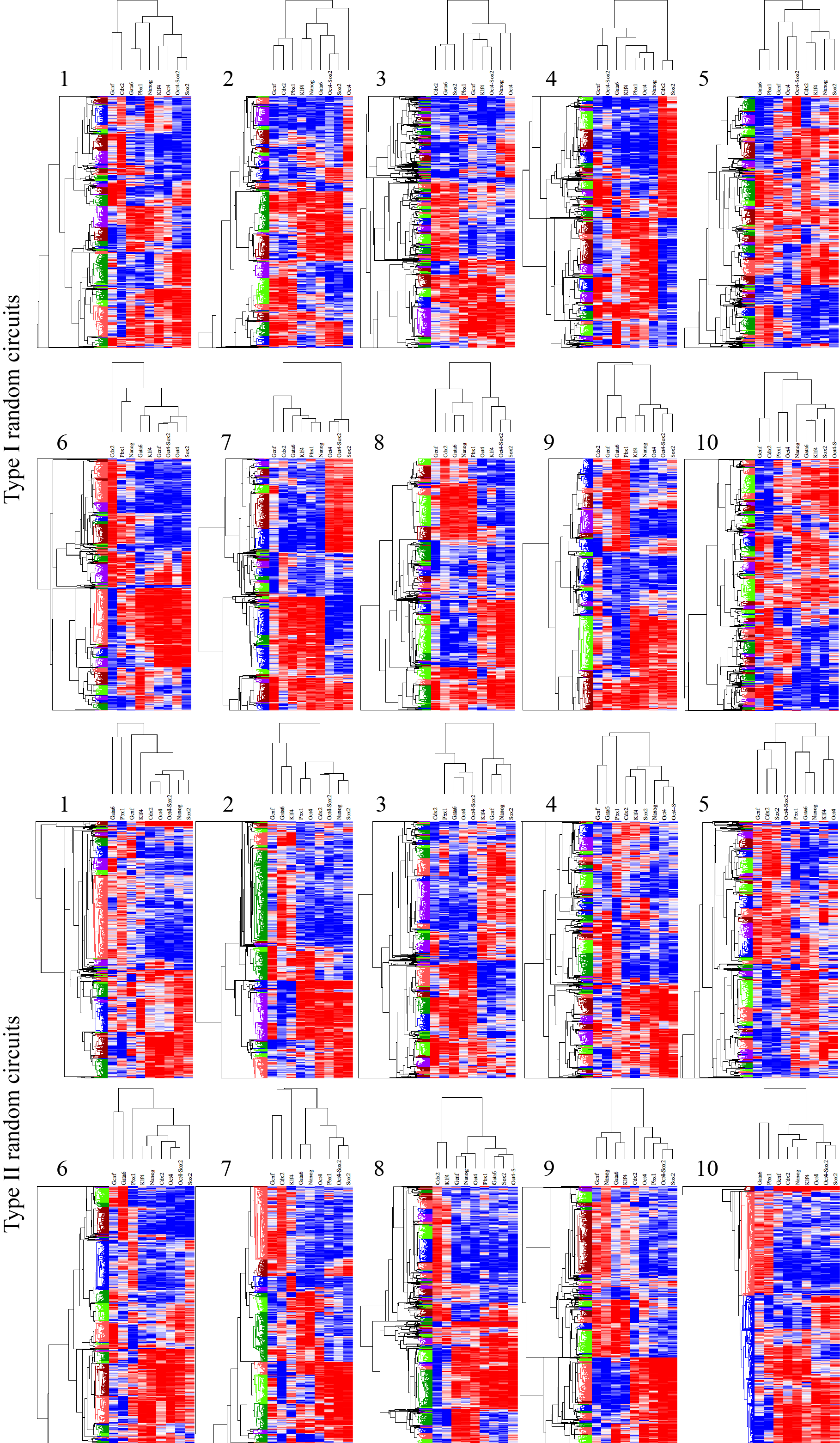


**Figure S12. Hierarchical clustering of the RACIPE gene expression data of random circuits.** Each column represents a gene and each row represents a steady state. The cutoff value for the clustering tree is set to be the same 0.8 as that of the stem cell networks. The identified major gene state clusters are highlighted by colors. (a) Type I random networks. (b) Type II random networks.

**­­­­­**

**Figure S13. Comparison between the RACIPE gene expression data of a random circuit and experimental gene expression data.** RACIPE was applied to each random circuit to generate a collection of gene expression data. (a)-(b) Percentage of the RACIPE predicted gene expression data in consistent with the experimental single cell gene expression data by the cluster-based analysis, where the average gene expression vectors were computed for each gene state cluster from both the RACIPE data and the experimental data. All the RACIPE data from a cluster was regarded to match the experimental data if the average gene expression vector match that of any experimental cluster. The comparison is performed for all experimental gene states (a) and also only the ones that belongs to the late stage (≥ 32 cells) of embryo development (b). (c) Percentage of the RACIPE predicted gene expression data in consistent with the experimental single cell gene expression data by the individual-based analysis. Instead of using the average gene expression vectors of predicted gene states, we performed direct one-on-one comparison between gene expression vector of each stable state of predicted data with each average gene expression vector of the experimental gene states (by MSEs). Blue circuits denote the type I random circuits, and red triangle denote the type II random circuits. The index in the x-axis is consistent with the order in **figure S9**.


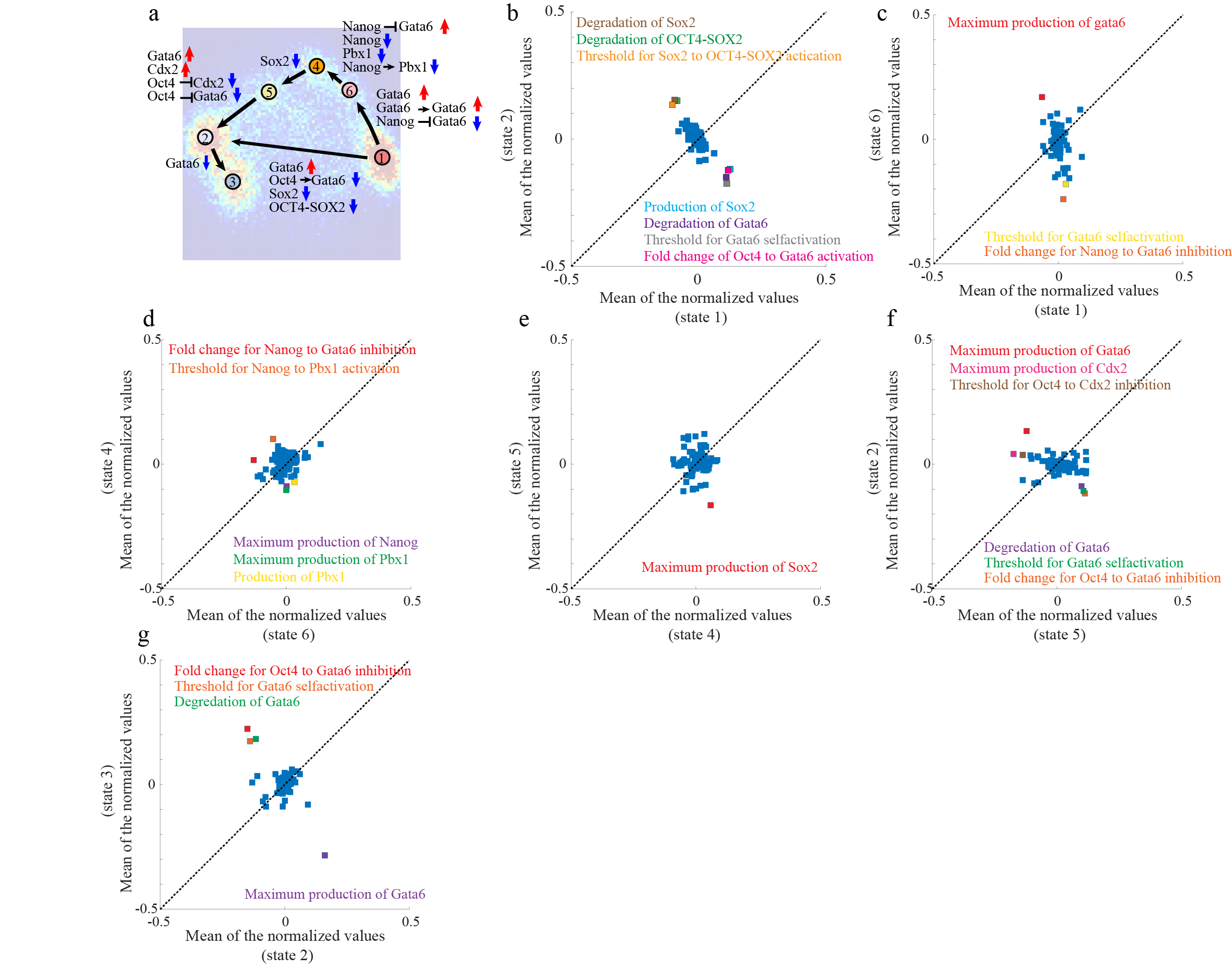


**Figure S14. Key parameters that are involved in the transitions among certain gene states.** (a) A summary of the results on top of the probability density map of the RACIPE predicted gene expression data. For each transition shown in panel (a), panels (b) to (g) show the mean of the normalized values for each parameter for the two corresponding gene states (x-axis for the first gene state, and y-axis for the second gene state). (b): states 1 and 2; (c): states 1 and 6; (d): states 6 and 4; (e): states 4 and 5; (f): states 5 and 2; (g): states 2 and 3.

**Figure S15. Behaviors of the sub-circuits Oct4/Cdx2 and Nanog/Gata6.** According to **figure 6**, there exist two sub-circuits (top layer: Oct4/Cdx2 and bottom layer: Nanog/Gata6) to form a hierarchical structure. (a) and (d): The RACIPE gene expression data of the full circuit are split into two sets, each of which corresponds to the genes from either the top sub-circuit (panel a) or the bottom sub-circuit (panel d). The hierarchical clustering results of them are shown in (a) and (d). (b), (c), (e) and (f): RACIPE was applied to each sub-circuit whose circuit parameters were randomized with the same ranges as those for the full circuit. The panels show the hierarchical clustering results for each RACIPE dataset. Panels b and e: for each gene, data are normalized by the mean and standard deviation of the expression levels for each sub-circuit; panels c and f: for each gene, data are normalized by the mean and standard deviation of the expression levels for the full circuit. The results show that the RACIPE results remain the same for the Oct4/Cdx2 sub-circuit when the regulatory links connecting the two sub-circuits are removed. However, the results for the Nanog/Gata6 sub-circuit are dramatically different no matter which normalization methods are used.


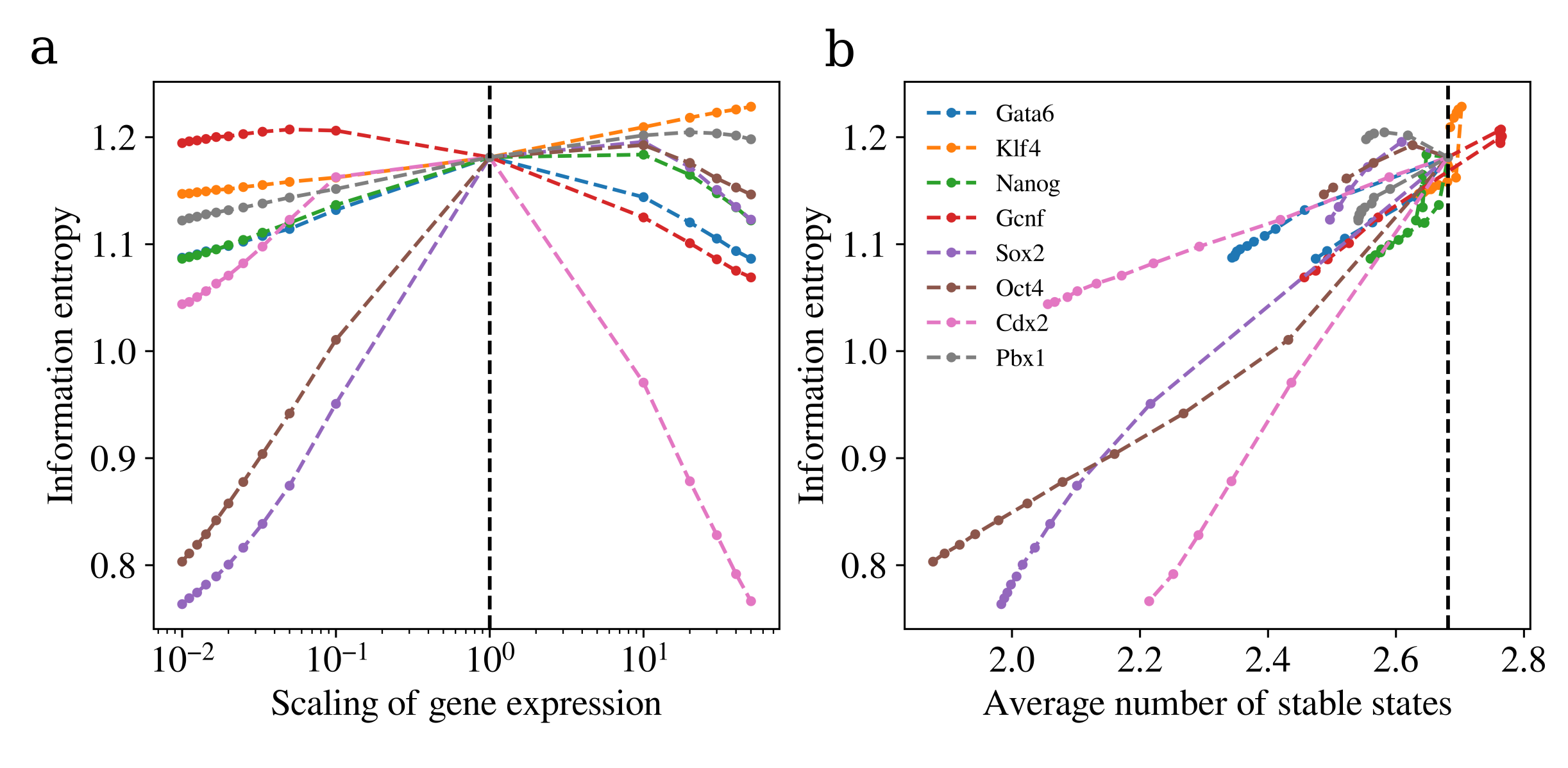


**Figure S16. Heterogeneity of the ensemble of the RACIPE models in response to low/high-expression treatments.** (a) Weighted (phylogenetic) information entropy as the function of the level of inhibition/activation for each gene. The scaling of gene expression represents the strength of external signals activating (larger than 10^0^) or inhibiting (smaller than 10^0^) a gene. The entropy is calculated using the method in **electronic supplementary material, §S11**. The gene expression profiles generated by 10,000 sets of parameters with no perturbation (referred to as the wide type (WT)) were used to perform the HCA to decide the gene state clusters and the branch lengths of each gene state cluster in formula (9). The gene expression profiles generated in each of the perturbation case were first classified into one of the gene state clusters identified in WT and then the entropy of that perturbation case can be calculated. As one set of parameters can generate multiple gene states. A weight factor proportional to the percentage of initial conditions leading to a gene state were multiplied to that gene state. (b) The information entropy is strongly correlated with the average number of stable states for each RACIPE model. The black dashed vertical lines represent the case without treatment.


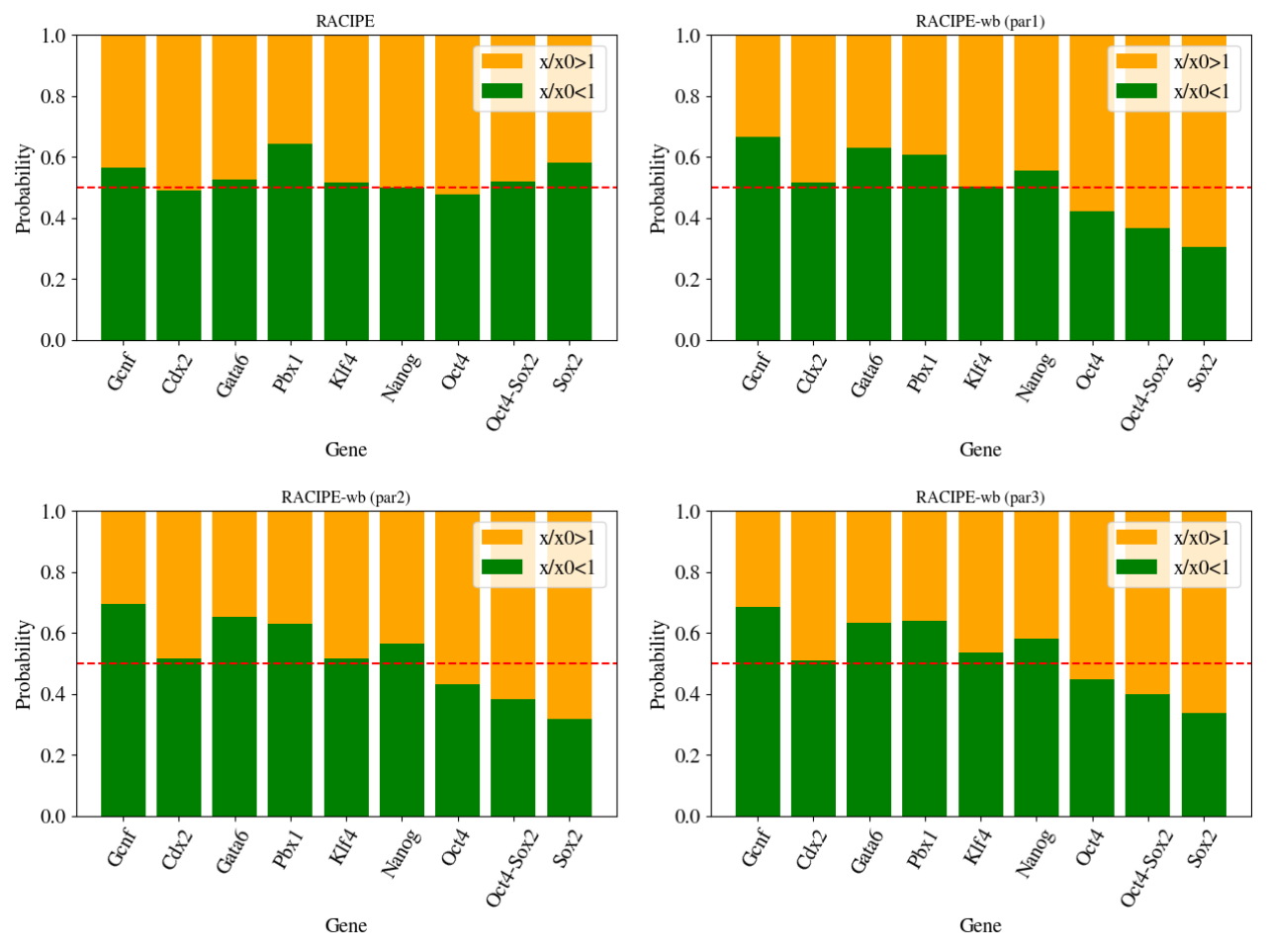
**Figure S17:** The probability that the results for a particular gene from the model result in a value greater than or equal to the estimated thresholds (listed in **Table S3**). The original RACIPE framework and the updated RACIPE-wb framework satisfy the half-functional rule.


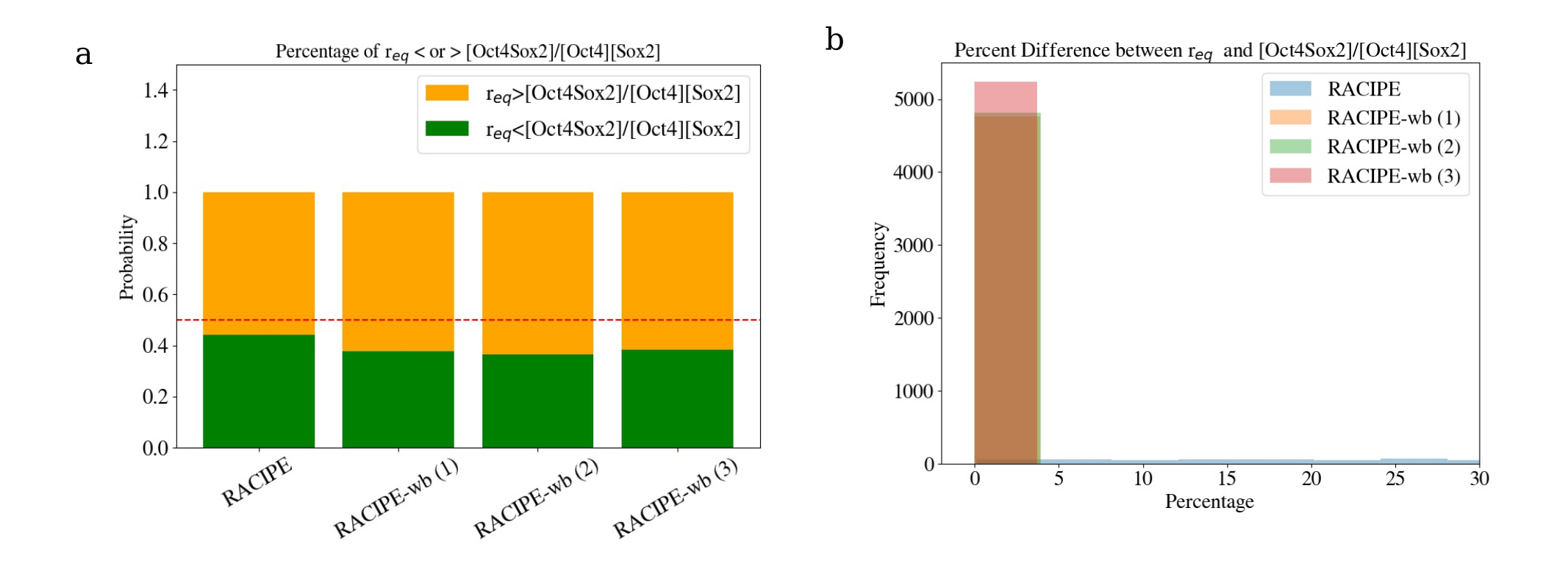


**Figure S18:** (a) The probability that the model results in an equilibrium state greater than or less than expected (i.e. greater than or less than *r_eq_* (*r_equilibrium_*) for the model based on the *r_unbinding_* and *r_binding_*). This is shown for the original RACIPE framework and for that with binding (RACIPE-wb) for 3 different ranges of *r_equilibrium_*. (b) The percent difference between the expected value of *r_equilibrium_* (given by the values in the model) and the actual *r_equilibrium_* value resulting from solving all 10000 models. Together (a) and (b) show that RACIPE and the 3 RACIPE-wb models approximately satisfy the half-functional rule.


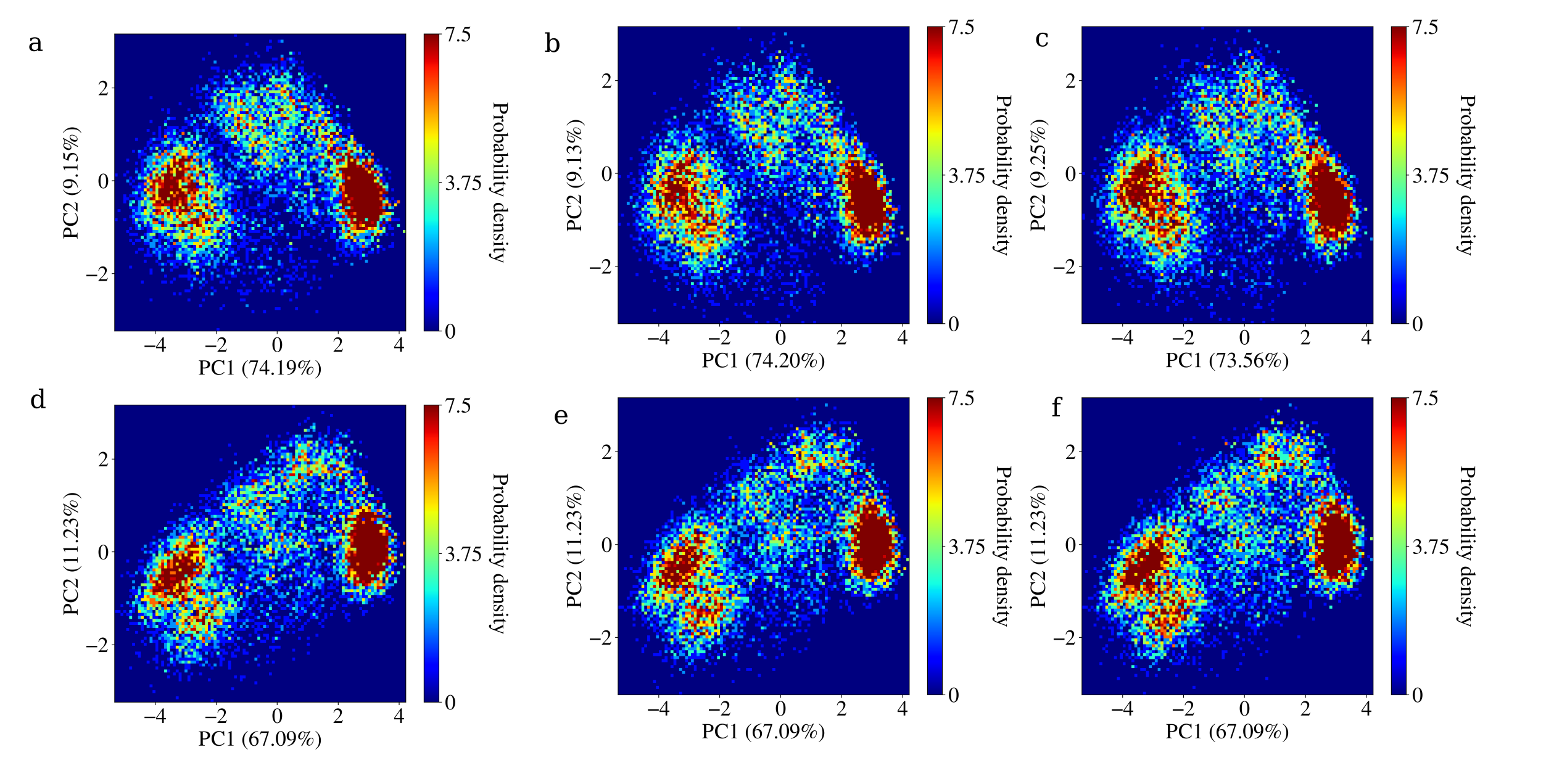
**Figure S19:** The probability density of RACIPE-wb (par1-par3) normalized by their respective z-scores and projected onto their own first and second PCA components, respectively (a-c). The probability density of RACIPE-wb (par1-par3) normalized by the z-score using the original RACIPE framework and projected onto the PCA components of the results from the original RACIPE framework (d-f).


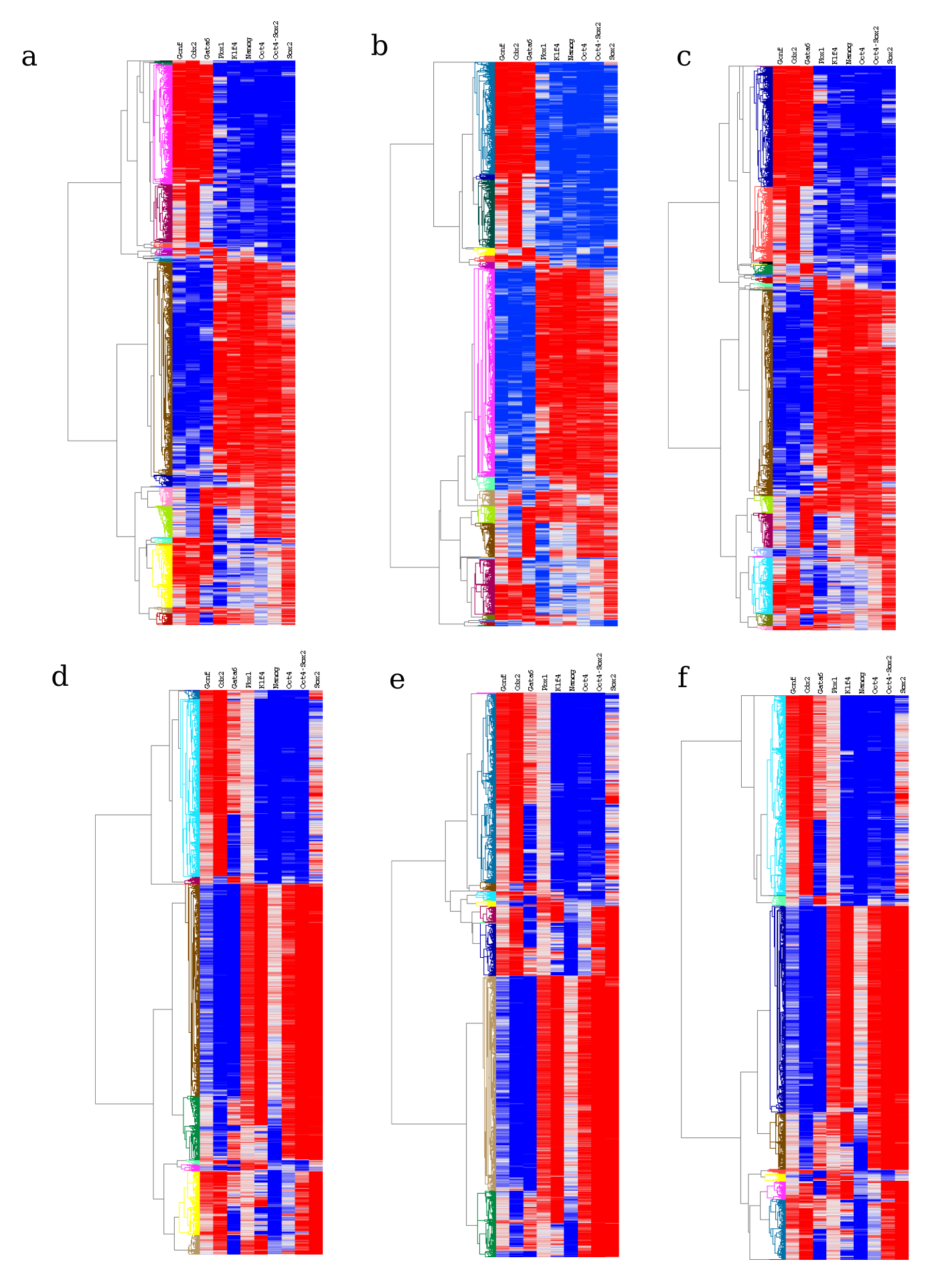


**Figures S20**: The results of RACIPE-wb (par1-par3), respectively, clustered using hierarchical clustering after normalization by their respective z-scores (a-c). The results of RACIPE-wb (par1-par3), respectively, normalized by the z-scores from the original RACIPE results and then clustered using HCA. For all plots, each distinctly colored region corresponds to a gene state (d-f).


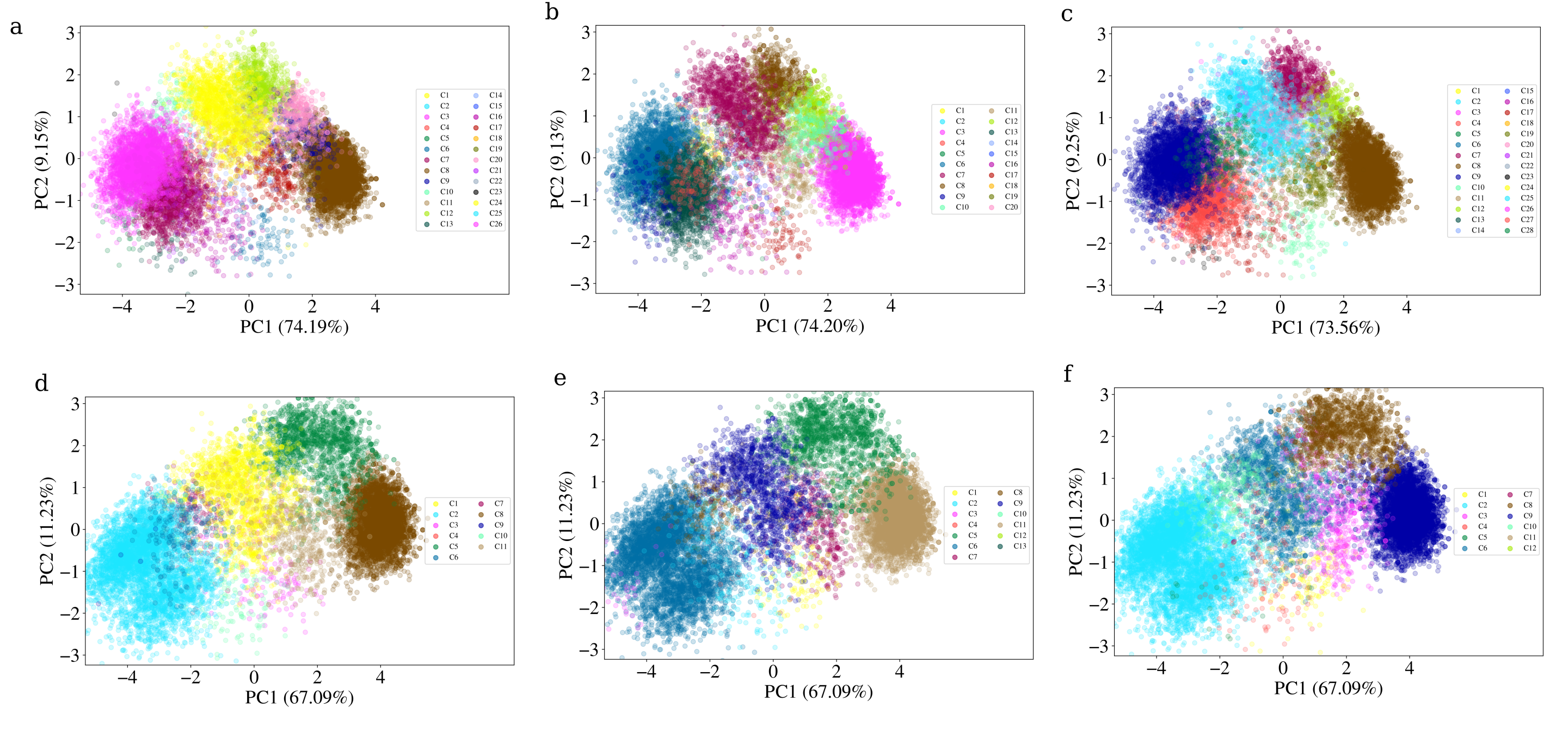
**Figure S21:** The clusters identified from HCA of par1-par3, respectively, projected onto their respective first and second PCA axes (a-c). The clusters identified from HCA of par1-par3 normalized by the z-scores of the original RACIPE results projected onto the first and second PCA axes of the results from the original PCA axes (d-f). The colors in these figures correspond to the clusters in **figure S20**.


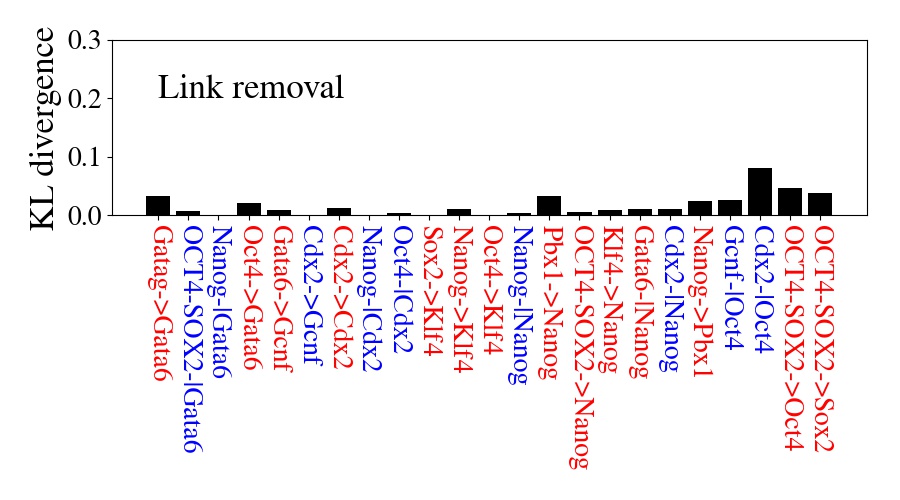


**Figure S22:** The KL divergence between the probability distributions of the number of stable states for each RACIPE-wb model computed before and after the removal of each regulatory link except for the removal of the binding/unbinding processes.
